# Hollow RuO_2_ nanozymes sensitized by carbon dot sonosensitizers for sonodynamic/chemodynamic-activated immunotherapy

**DOI:** 10.7150/thno.125880

**Published:** 2026-01-01

**Authors:** Ming Cao, Yanwei Liu, Zhenlin Zhang, Jinming Cai, Dengyu Pan, Bijiang Geng, Yunsheng Cheng

**Affiliations:** 1 Department of General Surgery, Second Affiliated Hospital of Anhui Medical University, Hefei, Anhui 230601, China.; 2 School of Environmental and Chemical Engineering, Shanghai University, Shanghai 200444, China.

**Keywords:** carbon dots, hollow RuO_2_ nanospheres, heterojunctions, sonodynamic therapy, immunotherapy

## Abstract

**Background:** Regulating the morphology structure of sonosensitizers and nanozymes is crucial to improve sonodynamic and enzyme-mimic activities.

**Methods:** We report for the first time the utilization of Cu_2_O nanospheres as the sacrificial templates for the synthesis of hollow RuO_2_ nanospheres (H-RuO_2_) for high-efficiency sonodynamic and chemodynamic therapy (SDT/CDT). We then utilized NIR phosphorescence carbon dots (CDs) as the auxiliary sonosensitizers to sensitize H-RuO_2_ for the construction of CD@H-RuO_2_ heterojunctions.

**Results:** Compared with solid nanoparticles, nanosheets, and other structures, the hollow RuO_2_ (H-RuO_2_) nanostructures are expected to exhibit stronger catalytic activity due to their larger specific surface area and more catalytic active sites. The improved electron-hole separation kinetics enable CD@H-RuO_2_ nanozymes with significantly enhanced sonodynamic and multienzyme-mimic activities. CD@H-RuO_2_-triggered cascade amplification of antitumor immune response was realized by the heterojunction construction, GSH depletion, and relief of hypoxia co-augmented ROS yield, which significantly induced a robust ICD.

**Conclusion:** CD@H-RuO_2_-mediated SDT and CDT co-amplified immunotherapy have shown significant antitumor effects, resulting in the eradication of primary tumors and the inhibition of distant tumor growth. This study offers hopeful insights into the fabrication of heterojunctions for sonodynamic/chemodynamic-activated immunotherapy

## Introduction

The treatment of colorectal cancer is highly challenging due to its high heterogeneity (diverse genetic mutations) and hidden risk of metastasis 1-3. To address colorectal cancer, a range of anticancer approaches like surgery, chemotherapy, radiotherapy, and targeted therapy can be used, but there is a critical need for more powerful strategies, particularly for advanced stages of the disease 4. Cancer immunotherapy has emerged as a promising approach for treating cancer. However, traditional chemotherapy and radiotherapy fail to generate a lasting immune response 5-8. In the treatment of colorectal cancer, the use of programmed death ligand 1 (PD-L1) inhibitors as part of immune checkpoint blockade (ICB)-mediated tumor therapy has increased 9-11. Nevertheless, due to low PD-L1 expression levels and impaired immune responses in the immunosuppressive tumor microenvironment (TME), a considerable number of patients have insufficient responses to ICB-induced tumor treatment 12-14. Additionally, patients receiving αPD-L1 monotherapy often develop different degrees of drug resistance or experience immune-related adverse reactions, ultimately affecting treatment outcomes 15-17. Given this situation, the development of innovative immunomodulatory strategies is essential to overcome the limitations of existing immunotherapy.

The emergence of immunogenic cell death (ICD) has opened up a new perspective for immunotherapy 18-21. However, the high toxicity and poor tolerance of chemotherapeutic drugs such as doxorubicin have limited their clinical application in inducing ICD 22, 23. Recently, various therapeutic strategies that use reactive oxygen species (ROS) to kill tumor cells and enhance immune responses have been developed, including sonodynamic, photodynamic, and chemodynamic therapy (SDT/PDT/CDT) 14, 24-29. Among them, sonodynamic therapy (SDT) has a higher penetration depth and can overcome the limitations of photodynamic therapy (PDT), effectively targeting tumors at different depths in the body 30-34. However, the limited ROS generation efficiency of sonosensitizers significantly restricts the effect of SDT-induced ICD. These sonosensitizers typically have wide bandgaps and rapid electron-hole recombination 35-40. Moreover, the intricate TME, defined by hypoxia and increased GSH expression, plays a crucial role in influencing the effectiveness of SDT 41-43. Hence, it is crucial to investigate high efficiency sonosensitizers and control immunosuppressive TME to increase ROS production and enhance ICD effectively.

Combining the SDT-triggered ROS production with other theranostic approaches that support ICD is necessary for a comprehensive activation of the immune response. CDT employs nanozymes for inducing endogenous catalytic reactions in TME, leading to the selective generation of ROS for cancer cell destruction, demonstrating both precise targeting and minimal toxicity 44-46. In addition, CDT can induce strong ICD without external energy, leading to an antitumor immune response and the ability to overcome drug resistance 47-49. Moreover, nanozymes exhibiting GSH-px-like and CAT-like activities can regulate the immunosuppressive TME and achieve cascade amplification of ROS production by decreasing the overexpressed GSH and increasing the production of O_2_ 50-52. However, the vast majority of nanozymes have low catalytic activity, and it is difficult to achieve complete eradication of tumors with a single CDT 53-57. Although some Cu-based nanozymes have high catalytic activity, the extreme toxicity of Cu^+^ often causes irreversible damage to normal tissues due to the inevitable leakage of nanodrugs 58, 59. In addition, previous reports have not yet focused on controlling the morphology of nanozymes to achieve rational regulation of their catalytic activity.

RuO_2_ has been widely used in fields such as biosensing, disease treatment, and environmental catalysis due to its excellent catalytic activity, high stability, and controllability 60, 61. Compared with the highly toxic Cu_2_O with uncontrolled ion leaching, RuO_2_ exhibited excellent biocompatibility 62. Compared with other widely studied metal oxide nanozymes such as manganese dioxide (MnO_2_) and cerium dioxide (CeO_2_), ruthenium dioxide (RuO_2_) has obvious advantages in *in vivo* therapeutic applications. Its excellent chemical stability significantly reduces the risk of toxic ion leakage, solving the potential long-term biosafety issues related to manganese or cerium ions. Furthermore, the mixed-valence states of Ru (Ru^3+^/Ru^4+^) endow it with multifaceted enzyme-mimicking activities, including POD-like and GSH-oxidase-like activities, which are crucial for synergistic CDT and TME regulation. In addition, its suitable bandgap structure makes it an ideal candidate for constructing heterojunctions to augment sonodynamic performance. Although the catalytic activity of RuO_2_ is weaker than that of unstable and easily oxidizable Cu_2_O, its good biocompatibility and stable catalytic activity make it more suitable for nanocatalytic reactions compared to Cu_2_O. Based on this situation, it is crucial to regulate the morphology and structure of RuO_2_ to enhance its catalytic activity. Compared with solid nanoparticles, nanosheets, and other structures, the hollow RuO_2_ (H-RuO_2_) nanostructures are expected to exhibit stronger catalytic activity due to their larger specific surface area and more catalytic active sites 61. Nevertheless, the use of H-RuO_2_ as nanozymes or sonosensitizers for CDT and SDT has not been previously reported.

In this work, we first utilized Cu_2_O nanospheres as the sacrificial templates for the synthesis of H-RuO_2_ nanospheres for high-efficiency CDT and SDT. The ingenious design of H-RuO_2_ structure is reflected in the choice of sacrificial templates. On the one hand, Cu_2_O is highly chemically reactive and prone to oxidation, indicating that its surface has many active sites that can efficiently adsorb and deposit metal ions. In contrast, the commonly used SiO_2_ templates require additional reducing agents or surface modifications to facilitate the deposition of metal oxides, leading to a more complex procedure and potential impurity introduction 63. On the other hand, the removal of Cu_2_O can be achieved through a mild etching with weakly alkaline ammonia water at room temperature within a short timeframe. Conversely, removing SiO_2_ often necessitates prolonged exposure (over 12 hours) to strong acids (e.g., HCl, HF) or bases (e.g., NaOH, KOH) under heating conditions 64, which may erode the deposited metal oxides due to the aggressive nature of these chemicals. On this basis, Cu_2_O nanospheres were selected as the templates for the synthesis of H-RuO_2_ nanospheres. As expected, the obtained H-RuO_2_ nanospheres exhibit good sonodynamic and multienzyme-mimic activities.

**Scheme 1:**
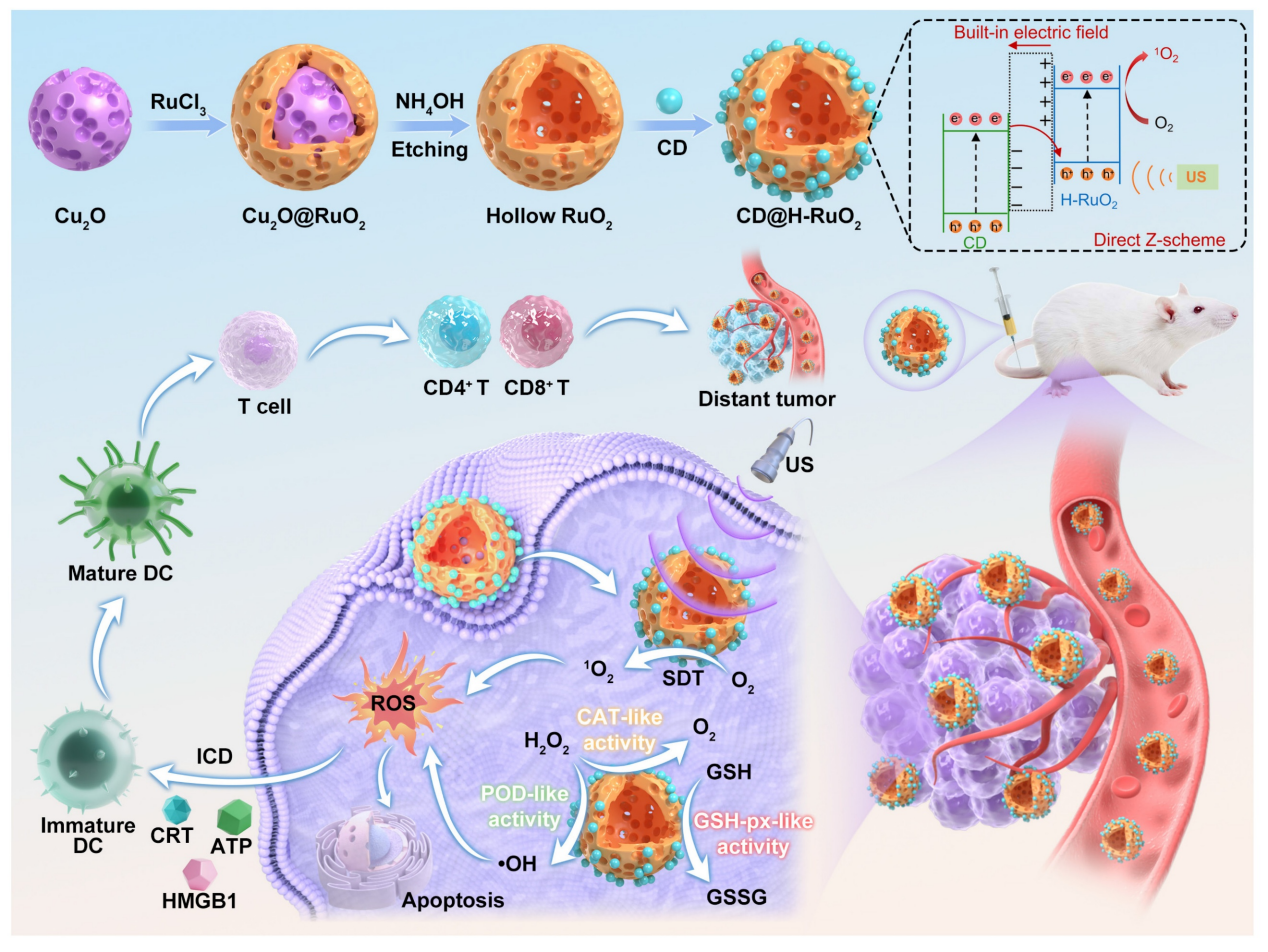
Schematic illustration of the preparation of CD@H-RuO_2_ heterojunctions for sonodynamic/chemodynamic-activated immunotherapy. Cu_2_O nanospheres were utilized as the sacrificial templates for the synthesis of H-RuO_2_, which was then sensitized by CD sonosensitizers for enhanced SDT and CDT.

To further improve the sonodynamic and multienzyme-mimic activities of H-RuO_2_, we utilized NIR phosphorescence CDs as the auxiliary sonosensitizers to sensitize H-RuO_2_ through coating CDs on H-RuO_2_. CD@H-RuO_2_ can act as heterojunction nanozymes and exhibit enhanced sonodynamic and multienzyme-mimic activities owing to the improved electron hole separation kinetics. Upon accumulation in tumor tissue, CD@H-RuO_2_ produced abundant ROS through the synergistic effect of SDT and CDT. The antitumor immune response triggered by CD@H-RuO_2_ can be further amplified from the following aspects. Firstly, the synergistic SDT and CDT through CD@H-RuO_2_ nanozymes can generate ROS for triggering ICD. Secondly, the SDT performance facilitated by CD@H-RuO_2_ was boosted even further by the reduction of hypoxia, resulting in an increased supply of O_2_ for SDT. Thirdly, the ROS-induced ICD may be intensified due to the increased ROS generation caused by the reduction of GSH. Satisfactory therapeutic outcomes for primary and distant tumors were obtained by CD@H-RuO_2_-mediated sonodynamic/chemodynamic-activated immunotherapy (Scheme 1).

## Results and Discussion

**Figure 1:**
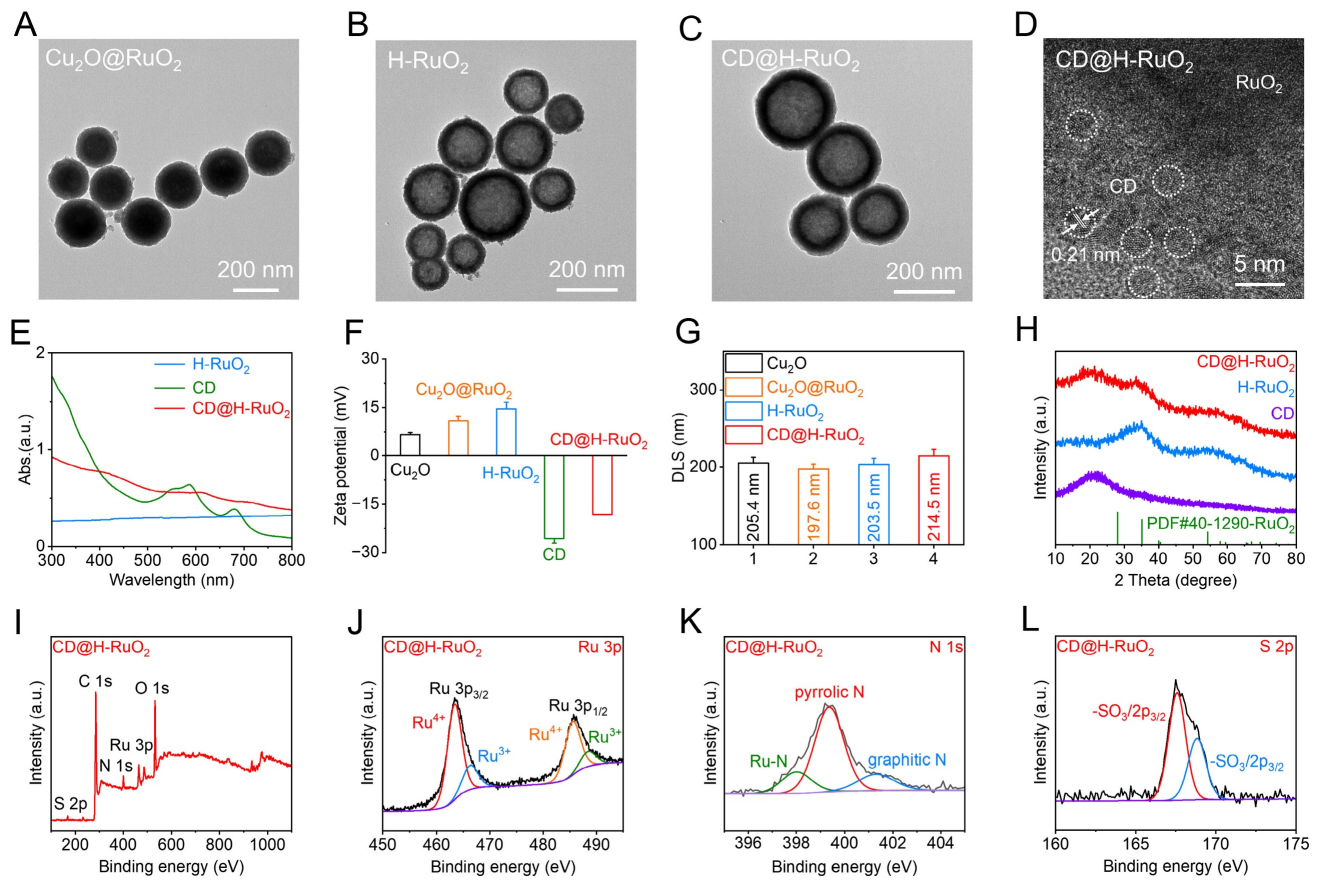
(A-C) TEM images of Cu_2_O@RuO_2_, H-RuO_2_, and CD@H-RuO_2_. (D) HRTEM of CD@H-RuO_2_. (E) Ultraviolet-visible absorption spectra of H-RuO_2_, CD and CD@H-RuO_2_. (F, G) Zeta potential and hydrodynamic diameter of Cu_2_O, Cu_2_O@RuO_2_, H-RuO_2_ and CD@H-RuO_2_. (H) XRD patterns of H-RuO_2_, CD and CD@H-RuO_2_. (I-L) Survey XPS (I), high-resolution Ru 3p (J), N 1s (K), O 1s (H), S 2p (L) spectra of CD@H-RuO_2_. Data are presented as the mean ± SD. (n = 3).

The synthesis process of the Z-type CD@H-RuO_2_ heterojunction was illustrated in Scheme 1. The Cu_2_O nanospheres were synthesized via a straightforward wet-chemical approach. TEM image of Cu_2_O nanospheres illustrated in Figure S1A revealed uniformly dispersed nanospheres with an average diameter of approximately 150 nm. High-resolution TEM image further indicated the lattice spacings of 0.254 nm and 0.207 nm, corresponding to the (200) and (111) crystal planes of Cu_2_O, respectively (Figure S1b). When Cu_2_O nanospheres are introduced into the RuCl_3_ solution, surface-exposed Cu^+^ ions promptly donate electrons to Ru^3+^ in solution. The reduced Ru species subsequently undergo hydrolysis or oxidation at the Cu_2_O interface, forming hydrated RuO_2_ (RuO_2_·xH_2_O), which deposits epitaxially to build a continuous shell. This transformation initiates at the outer surface of the Cu_2_O spheres and progresses outward, ultimately yielding a well-defined RuO_2_ shell. As shown in Figure 1A, the RuO_2_ shell is uniformly coated on the surface of Cu_2_O. Due to the partial etching of Cu_2_O during the deposition process, its size change is extremely small. The subtle size change of Cu_2_O@RuO_2_ compared with the original Cu_2_O was further confirmed by DLS measurements (Figure 1G), indicating that the hydrodynamic diameters of Cu_2_O@RuO_2_ and Cu_2_O were determined to be 197.6 nm and 205.4 nm, respectively. We also detected the XRD patterns and XPS spectra of Cu_2_O@RuO_2_ and Cu_2_O. As depicted in Figure S2, no significant change of diffraction peaks can be detected in Cu_2_O core before and after RuO_2_ shell deposition. The absence of RuO_2_-related peaks in the XRD pattern can be attributed to the relatively low synthesis temperature (80°C), which resulted in an amorphous/defective nature of H-RuO_2_. The survey XPS spectrum depicted in Figure S3A revealed the presence of Cu and Ru, demonstrating the successful coating of RuO_2_ shell. We also detected the existence of Ru^4+^, Ru^3+^, Cu^+^, Cu^2+^, and O-H in Cu_2_O@RuO_2_ (Figures S3B-D).

Subsequently, the Cu_2_O core was selectively removed using ammonia water etching method, yielding the hollow RuO_2_ structure. The TEM image revealed the hollow structure of H-RuO_2_ (Figure 1B). The poor crystalline of H-RuO_2_ was then verified by the XRD pattern (Figure 1H). By comparing with the standard PDF card of RuO_2_, we found that the diffraction peak at 35° and 55° could be attributed to RuO_2_. However, it presented a very broad diffraction peak, indicating that H-RuO_2_ exhibits an amorphous nanocrystalline structure. Before and after Cu_2_O etching, the size of H-RuO_2_ did not exhibit obvious changes (Figure 1F). After etching, Ru^4+^, Ru^3+^, and O-H were also presented in the XPS spectrum of H-RuO_2_ (Figure S4), suggesting the successful preparation of H-RuO_2_. We also found the positively charged features of H-RuO_2_, suggesting that H-RuO_2_ could bind to nanomaterials with negatively charged characteristics.

We then synthesized negatively charged CDs with long-lived triplet state through a simple microwave method according to our previous reports 40, 65. A lattice spacing of 0.21 nm was revealed in the high-resolution TEM image, demonstrating the successful preparation of CDs (Figure S5). XRD pattern presented in Figure 1H also verified the successful synthesis of CDs, which exhibited a broad diffraction peak at approximately 22°. XPS spectrum illustrated in Figure S6 indicated that CDs contained sulfonic acid group, which endowed CDs with negatively charged features. The negatively charged characteristics of CDs were confirmed by the Zeta potential measurements (Figure 1F). In addition, the presence of pyrrolic N in CDs was also demonstrated by the XPS measurements, which suggested that the possible binding mode between CDs and H-RuO_2_ was Ru-N coordination bond.

Finally, the negatively charged CDs were electrostatically adsorbed onto the positively charged H-RuO_2_ surface, forming the Z-type CD@H-RuO_2_ heterojunctions. A series of characterization experiments were conducted to confirm the successful synthesis of CD@H-RuO_2_. TEM image indicated that the surface of H-RuO_2_ was coated with CDs (Figure 1C), which also indicated that the size of CD@H-RuO_2_ was slightly larger than that of the pristine H-RuO_2_. The increased size of CD@H-RuO_2_ compared with H-RuO_2_ was also demonstrated by the DLS measurements (Figure 1G). High-resolution TEM image of CD@H-RuO_2_ revealed a lattice spacing of 0.21 nm corresponding to CDs (Figure 1D), while no distinct lattice fringes associated with H-RuO_2_ were observed, suggesting its amorphous/defective crystalline structure. Furthermore, through the EDS element mapping analysis, it was further determined that CD@H-RuO_2_ is composed of Cu (3.51 at.%), Ru (19.63 at.%), N (6.87 at.%), O (68.00 at.%) and S (1.99 at.%). The low copper content indicates that Cu_2_O has been almost completely etched. (Figure S7). UV-vis absorption spectroscopy exhibited that H-RuO_2_ possessed a flat horizontal absorption profile, whereas after loading CDs, the absorption spectrum underwent significant alterations (Figure 1E). Furthermore, as shown in Figure 1F, the Zeta potential of positively charged H-RuO2 becomes negative after loading carbon dots. XRD analysis further confirmed the structural evolution during synthesis. In the XRD pattern of CD@H-RuO_2_ (Figure 1H), a diffraction peak appeared at approximately 22°, corresponding to the [002] crystal plane of the graphite-like structure of CDs. As shown in Figure 1I, CD@H-RuO_2_ was primarily composed of five elements: C, N, O, S, and Ru. The XPS signal of Ru 3p was deconvoluted into four peaks: two peaks located at 463.5 eV and 485.8 eV correspond to Ru^4+^, while the other two peaks at 466.4 eV and 488.5 eV are associated with Ru^3+^ (Figure 1J). Nitrogen in the forms of pyrrole nitrogen and graphite nitrogen are the predominant forms in the N 1s spectrum of CD@H-RuO_2_, furthermore, the new peak fitted at 397.8 eV can be attributed to the Ru-N bond (Figure 1K), whereas sulfur mainly exists as sulfonic acid groups and the several peaks fitted in the C1s XPS can be respectively attributed to C-C/C=C, C-O, C-N and C-S bonds (Figures 1L and S8). Collectively, these results confirmed the successful loading of CDs onto the surface of H-RuO_2_. CD@H-RuO_2_, CDs, and H-RuO_2_ were placed in PBS and RPMI-1640 culture medium, and their behaviors over different time periods were monitored. As presented in Figure S9, the DLS results revealed that the size of CD@H-RuO_2_ remained nearly unchanged after storage for various durations in either PBS or RPMI-1640 solution, demonstrating its good colloidal stability. In contrast, H-RuO_2_ exhibited a gradual increase in size after storing in PBS or RPMI-1640 solution, which could be ascribed to the aggregation of H-RuO_2_. The enhanced stability of CD@H-RuO_2_ can be attributed to the excellent biocompatibility of CDs, which formed a stable surface modification layer on H-RuO_2_. The hydrophilic functional groups on the surface of CDs substantially improved the colloidal stability of the composite material.

**Figure 2:**
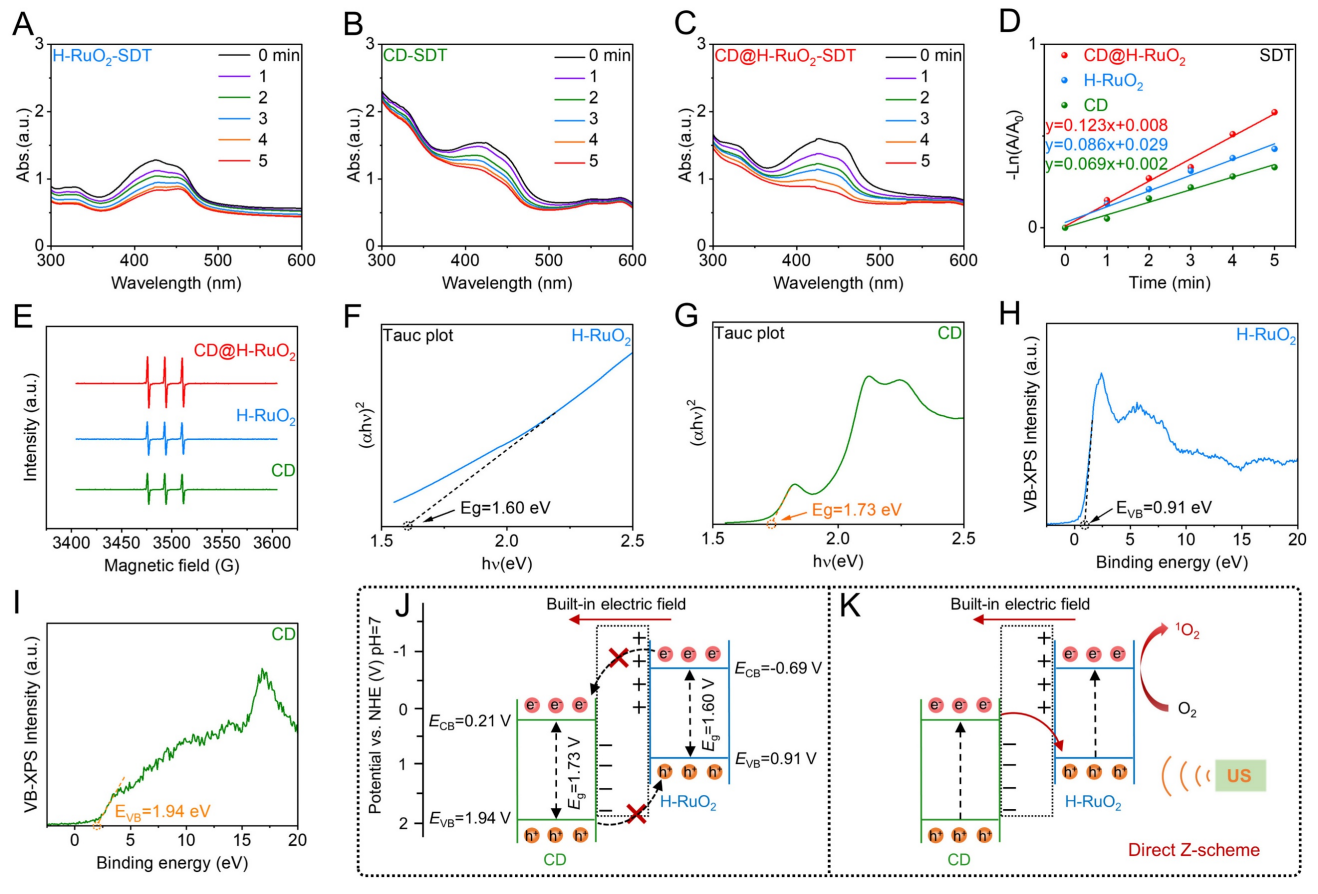
(A-C) Ultraviolet-visible absorption spectra of DPBF indicated the ROS generation capability of H-RuO_2_, CD and CD@H-RuO_2_. (D) Quantitative fitting of reactive oxygen species generation rates of H-RuO_2_, CD and CD@H-RuO_2_. (E) Detection of Singlet Oxygen Generation from H-RuO_2_, CD and CD@H-RuO_2_ by ESR Spectroscopy. (F-I) Determination of the bandgap and valence band spectrum of H-RuO_2_ and CDs. (J, K) Illustrations of the energy band diagrams of the Z-type CD@H-RuO_2_.

After confirming the successful preparation of the CD@H-RuO_2_ Z-type heterojunctions, we further investigated their amplified sonodynamic activity. To evaluate the generation of ROS, we employed DPBF as a ROS probe. We evaluated the sonodynamic activity of CD@H-RuO_2_ prepared at different mass ratio of CD to H-RuO_2_. We found that the highest sonodynamic activity of CD@H-RuO_2_ can be achieved at the mass ratio of CD to H-RuO_2_ of 2 : 1 (Figure S10). Further increasing the mass ratio to 4 : 1 resulted in negligible enhancement in sonodynamic activity, likely because the adsorption capacity of H-RuO_2_ toward CDs had already been saturated. Compared with H-RuO_2_ without CDs deposition (Figure 2A) and CDs alone (Figure 2B), CD@H-RuO_2_ exhibited significantly enhanced ROS production under US irradiation (Figure 2C). The degradation rate constant of DPBF for CD@H-RuO_2_ was found to be 0.123 min^-1^, surpassing that of H-RuO_2_ (0.086 min^-1^) by 1.43 times and that of CDs alone (0.069 min^-1^) by 1.78 times (Figure 2D). These results indicated that the construction of the Z-type heterojunctions effectively suppressed the recombination of electron-hole pairs. To further confirm the generation of ROS, ESR measurements were conducted using TEMP as the ^1^O_2_ scavenger to detect the ROS production capacity of CD@H-RuO_2_, H-RuO_2_, and CDs. As shown in Figure 2E, the ESR signal intensity of CD@H-RuO_2_ is stronger than that of H-RuO_2_ and CDs alone. Therefore, the incorporation of CDs into H-RuO_2_ enhances its sonodynamic performance, which may be attributed to the enhanced carrier transfer within the Z-scheme heterojunction.

To clarify the potential mechanism of the enhanced sonodynamic performance of the CD@H-RuO_2_ Z-scheme heterojunction, Firstly, electrochemical impedance spectroscopy (EIS) measurements were conducted on H-RuO_2_, CD and CD@H-RuO_2_. As shown in Figure S11, the charge transfer resistance of the CD@H-RuO_2_ Z-scheme heterojunction is lower than that of each individual component, indicating that the construction of heterojunction enhanced the interfacial charge transfer efficiency. Next, we calculated the band gap widths of H-RuO_2_ and CDs, which were 1.60 eV (Figure 2F) and 1.73 eV (Figure 2G), respectively. Additionally, XPS-VB analysis revealed that the valence band position of H-RuO_2_ was 0.91 eV (Figure 2H), while that of CDs was 1.94 eV (Figure 2I). Based on these data, we constructed the band alignment diagram of the CD@H-RuO_2_ heterojunction (Figure 2J). Considering the surface charge characteristics, an internal electric field prevented electron transfer from positively charged H-RuO_2_ to negatively charged CDs. Conversely, the internal electric field assists in the electron transfer from the conduction band of CDs to the valence band of H-RuO_2_, confirming the formation of a Z-type heterojunctions in the CD@H-RuO_2_ composites (Figure 2K). Such interfacial charge transfer significantly prolonged the carrier lifetime and inhibits electron-hole recombination. From a thermodynamic perspective, the CB potential of H-RuO_2_ (-0.69 eV) is much more negative than the O_2_/O_2_- redox potential (-0.33 V vs. NHE), favoring the efficient generation of ROS.

**Figure 3:**
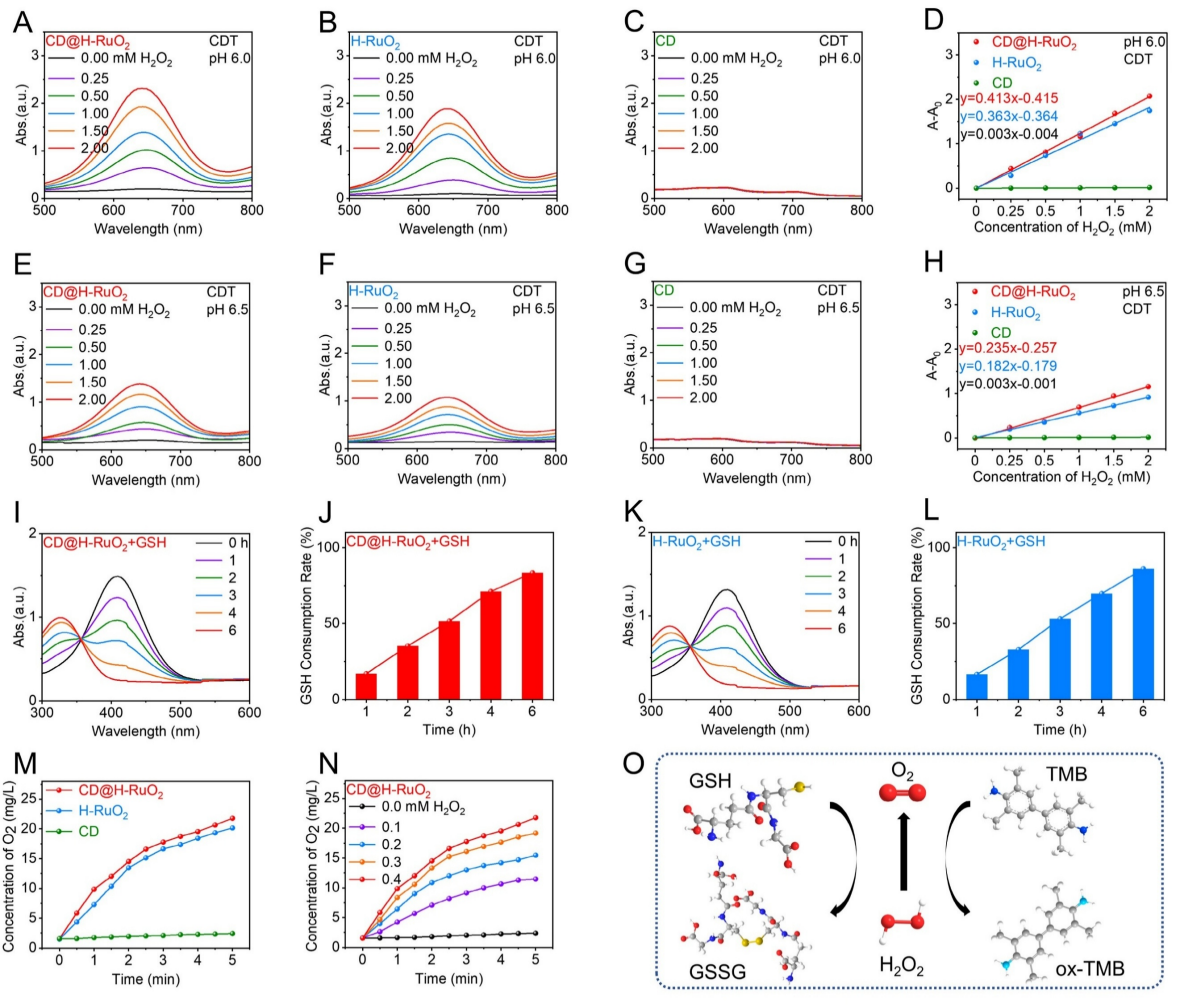
(A-D) Ultraviolet-visible absorption spectrum and quantitative fitting indicated the CDT activity of CD@H-RuO_2_, H-RuO_2_ and CD at pH 6.0. (E-H) Ultraviolet-visible absorption spectrum and quantitative fitting indicated the CDT activity of CD@H-RuO_2_, H-RuO_2_ and CD at pH 6.5. (I-L) Measurements of GSH consumption of CD@H-RuO_2_ and H-RuO_2_. (M, N) Evaluation of O_2_ production of CD@H-RuO_2_ and H-RuO_2_. (O) Schematic diagram of catalytic reactions of CD@H-RuO_2_.

Given the presence of Ru^3+^ in CD@H-RuO_2_, we employed TMB as a probe to investigate the CDT activity of the Z-type heterojunctions of CD@H-RuO_2_ based on the catalytic effect of Ru^3+^ on the Fenton-like reaction of H_2_O_2_. At pH 6.0, as the concentration of H_2_O_2_ increased, the absorbance at 652 nm for both CD@H-RuO_2_ and H-RuO_2_ significantly rose (Figures 3A, B). CD@H-RuO_2_ showed a rate constant of 0.413 min^-1^ for •OH generation, while H-RuO_2_ exhibited a rate constant of 0.363 min^-1^ (Figure 3D), indicating that CD@H-RuO_2_ exhibited the higher Fenton-like catalytic activity. This is attributed to the heterojunction structure, which facilitates efficient separation and directional migration of electron-hole pairs via an interfacial built-in electric field, thereby effectively suppressing charge recombination and increasing the availability of reactive electrons for peroxidase-like or catalase-like reactions. Meanwhile, the interface electronic reconstruction optimizes the adsorption energy of reaction intermediates, enabling the complementary catalytic functions of multiple components to be coordinated and exposing more active sites. These combined effects enhance catalytic efficiency 68, 69. Additionally, the enzyme catalytic rate constant further confirms this. As shown in Figure S12, the calculated *V*max of CD@H-RuO_2_ is 2.32×10-7 M s^-1^, slightly higher than that of H-RuO_2_ at 2.22×10-7 M s^-1^, while its *K*m value is 3.69 mM, slightly lower than that of H-RuO_2_ at 4.16 mM. These kinetic parameters indicate an improvement in both substrate affinity and catalytic efficiency, suggesting superior enzymatic activity. Moreover, a comparative analysis with previously reported nanozymes further validates the outstanding peroxidase-like activity of CD@H-RuO_2_ (Table S1). In contrast, under the same conditions, CDs showed no POD activity (Figure 3C). At pH 6.5, the hydroxyl radical generation rates of CD@H-RuO_2_ (Figure 3E) and H-RuO_2_ (Figure 3F) were both lower than those at pH 6.0 (Figure 3H). Notably, no obvious POD activity was observed for the three materials at pH 7.4 (Figure S13), indicating that CD@H-RuO_2_ and H-RuO_2_ would not exhibit toxicity to normal tissues with physiological pH values. Furthermore, the superior CDT activity of CD@H-RuO_2_ and H-RuO_2_ was further confirmed by ESR spectra using DMPO as a hydroxyl radical scavenger (Figure S14). These results collectively demonstrate that the CDT activity of CD@H-RuO_2_ and H-RuO_2_ has pH-responsive characteristics, highlighting their potential for tumor-specific therapeutic applications.

Furthermore, we investigated the ability of CD-H-RuO_2_ to consume GSH. After incubation with GSH for different periods of time, the characteristic absorption peaks of DTNB in CD@H-RuO_2_ (Figure 3I) and H-RuO_2_ solution (Figure 3K) were significantly reduced, indicating that these materials could effectively consume GSH. In contrast, pure CDs did not have the ability to consume GSH (Figure S15). After 6 hours of incubation, CD@H-RuO_2_ and H-RuO_2_ almost consumed all of the GSH (Figures 3J, L), which indicated that CD@H-RuO_2_ had a stronger ability to consume GSH, thereby enhancing the efficiency of ROS generation. In addition, oxygen plays a crucial role in SDT as they contribute to the generation of ROS. Therefore, we investigated the ability of CD@H-RuO_2_ to catalyze the generation of oxygen from H_2_O_2_. As shown in Figure 3M, the oxygen generation capacity of CD@H-RuO_2_ and H-RuO_2_ is comparable, while CDs exhibit almost no oxygen generation activity. As depicted in Figure 3N, the amount of oxygen generated gradually increases with the increase in H_2_O_2_ concentration, confirming its dependence on H_2_O_2_. These results collectively indicate that the CD@H-RuO_2_ heterojunction exhibits excellent TME-responsive properties. It can effectively utilize the excessive H_2_O_2_ in the TME to generate highly cytotoxic •OH radicals, while simultaneously generating oxygen to alleviate the hypoxic condition of tumors, thereby enhancing the efficacy of SDT. Additionally, its superior glutathione depletion performance prevents the excessive clearance of ROS, thereby maximizing the amplification effect of ROS and therapeutic efficacy (Figure 3O).

**Figure 4:**
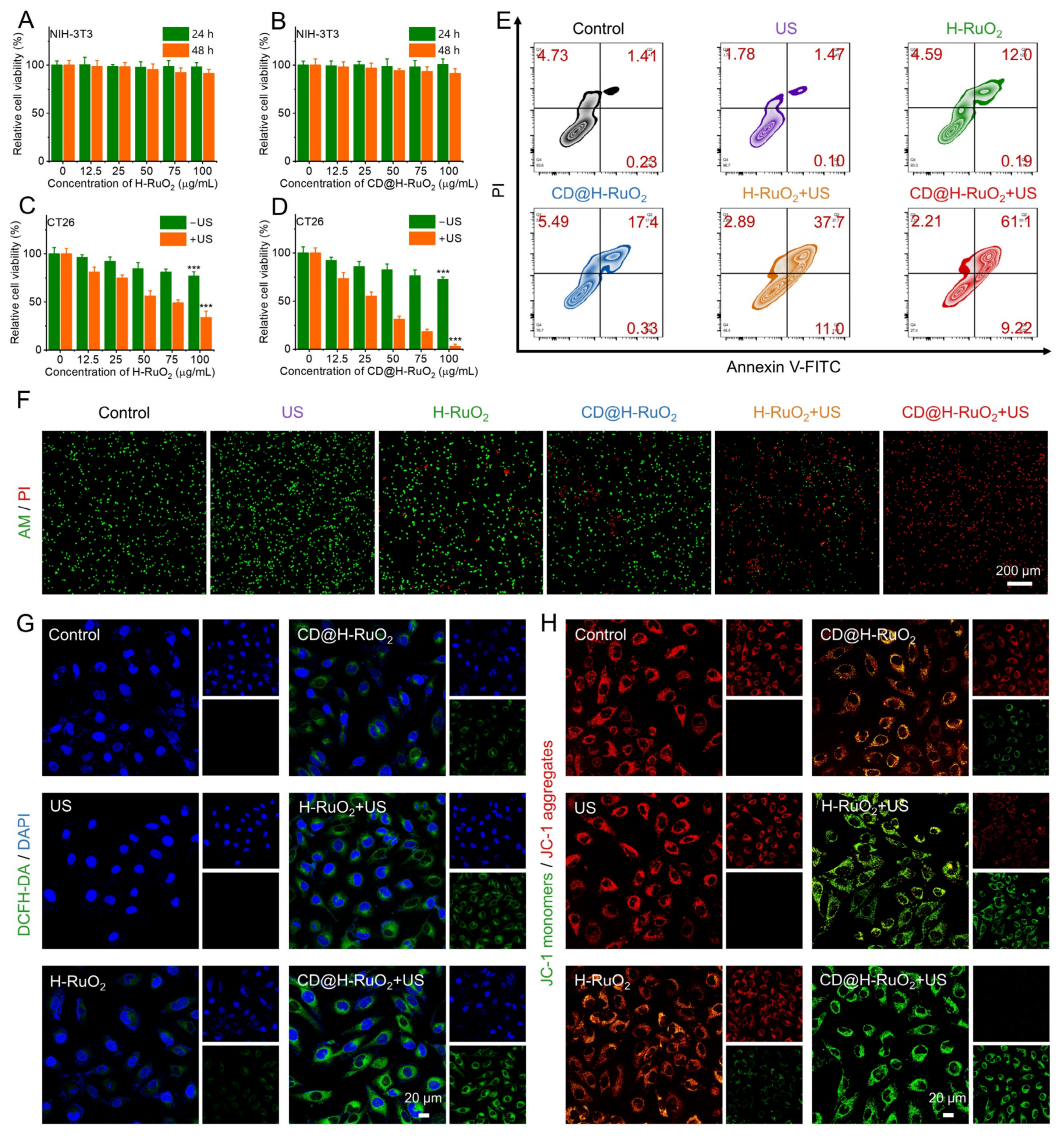
(A, B) Measurements of the cytotoxicity of H-RuO_2_ and CD@H-RuO_2_ against NIH-3T3. (C, D) Measurements of the cytotoxicity of H-RuO_2_ and CD@H-RuO_2_ against CT26 with or without US. (E-H) Apoptosis assay, live/dead and ROS staining, and mitochondrial membrane potential assay of CT26 cells after different treatments. Data are presented as the mean ± SD. (n = 6). ***p < 0.001.

Based on the excellent sonodynamic and chemodynamic activities of CD@H-RuO_2_, we assessed the combined antitumor effects of CD@H-RuO_2_ in SDT/CDT therapy at the cellular level. Mouse embryonic fibroblast NIH-3T3 and mouse colorectal cancer CT-26 cells were used as cellular models. Different concentrations of CD@H-RuO_2_, H-RuO_2_, and CD were incubated with NIH-3T3 cells, and the cell survival rate remained consistently above 90%. This phenomenon indicated that CD@H-RuO_2_, H-RuO_2_, and CD exhibited good biocompatibility (Figures 4A, B, and S16A). To evaluate the antitumor efficacy of the combined SDT and CDT treatment mediated by the Z-type heterojunction of CD@H-RuO_2_, cytotoxicity experiments were conducted using CT-26 cells. Regarding the single CDT-mediated antitumor effects via CD@H-RuO_2_ and H-RuO_2_, both materials exhibited low cytotoxicity when used alone to stimulate CT26 cells. Even at a concentration of 100 μg/mL, the survival rates of cancer cells were 76.9% for H-RuO_2_ and 72.5% for CD@H-RuO_2_, indicating that single CDT treatment was insufficient to completely eradicate cancer cells. In contrast, CD alone showed negligible toxic side effects on CT-26 cells (Figure S16B). For the *in vitro* synergistic CDT-SDT treatment effect of CD@H-RuO_2_, after US irradiation, the survival rates of CT-26 cells treated with H-RuO_2_ and CD@H-RuO_2_ were significantly reduced. Specifically, the survival rate of CT-26 cells decreased to approximately 33.9% after H-RuO_2_+US treatment (Figure 4C), while it dropped dramatically to 3.13% after CD@H-RuO_2_+US treatment (Figure 4D). To demonstrate the synergistic effect between CDT and SDT, we utilized mannitol to scavenge •OH generated by CDT. As illustrated in Figure S17, the "CD@H-RuO_2_ + mannitol" group represented no SDT and CDT. The "CD@H-RuO_2_" represented CDT alone. The "CD@H-RuO_2_ + mannitol + US" group represented SDT alone. The "CD@H-RuO_2_ + US" group represented the combined SDT and CDT. We then utilized MTT assay to evaluate the cell viability of CT26 cells after different treatments. The results indicated that CD@H-RuO_2_ exhibited negligible cytotoxicity against CT26 cells in the no SDT/CDT group. In contrast, CDT alone and SDT alone induced 28% and 67% cell death, respectively. Notably, when CDT and SDT were combined, CD@H-RuO_2_ achieved nearly complete (almost 100%) elimination of CT26 cells. Additionally, we conducted live/dead staining experiments to compare six different treatment methods: control, US, H-RuO_2_, CD@H-RuO_2_, H-RuO_2_ + US, and CD@H-RuO_2_ + US. As shown in Figure 4F, the proportion of dead cells in the CD@RuO_2_ + US group was significantly higher than in the other groups.

Then, we employed the DCFH-DA probe to stain CT-26 cells after different treatments to evaluate intracellular ROS levels. Figure 4G demonstrated that there was no significant green fluorescence signal in CT-26 cells treated with PBS or US alone. On the other hand, there was a slight green fluorescence indicating ROS generation in the H-RuO_2_ and CD@H-RuO_2_ groups, possibly due to the Ru^3+^-mediated Fenton-like catalytic reaction. Furthermore, when exposed to US irradiation, the CD@H-RuO_2_ Z-type heterojunction showed a notably stronger green fluorescence signal in comparison to the pristine H-RuO_2_, further demonstrating that the construction of the Z-type heterojunction enhanced ROS generation. In addition, extensive studies have shown that excessive ROS can lead to mitochondrial damage and apoptosis. Mitochondrial membrane potential (MMP) is highly sensitive to the accumulation of ROS in mitochondria. We used JC-1 as a probe to detect the changes in MMP in CT26 cells after six different treatments. The shift from red fluorescence to green fluorescence is a sign of mitochondrial dysfunction. According to confocal images (Figure 4H), the CD@H-RuO_2_ + US group showed the strongest green fluorescence and the weakest red fluorescence, indicating severe mitochondrial damage. Flow cytometry analysis further revealed that the total apoptosis rate of CT26 cells increased from 6.37% in the control group to 72.53% in the CD@H-RuO_2_ + US group (Figure 4E). In conclusion, these results indicate that CD@H-RuO_2_ has excellent *in vitro* anti-tumor performance.

**Figure 5:**
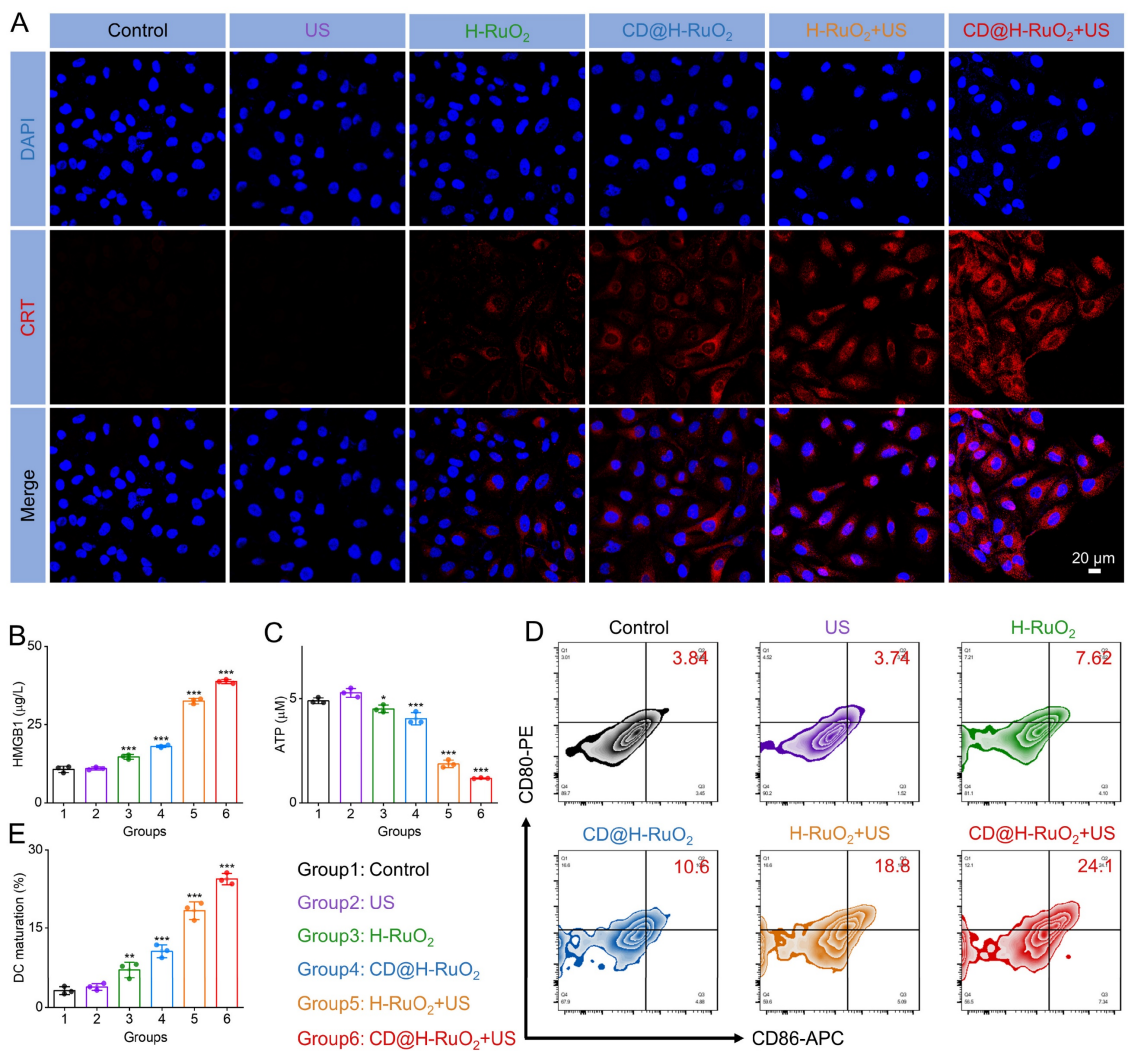
(A) Detection of CRT expression in CT26 cells after different treatments by immunofluorescence staining. (B, C) Measurements of HMGB1 and ATP levels in CT26 cells after different treatments. (D, E) The expression of CD80 and CD86 in DCs after different treatments determined by flow cytometry. Data are presented as the mean ± SD. (n = 3). *p < 0.05, **p < 0.01, and ***p < 0.001.

Furthermore, existing studies have demonstrated that the generation of ROS can effectively trigger ICD 21-23, 66. During ICD, dying cells release DAMPs, facilitating the engulfment of tumor cells by APCs, thereby promoting tumor-specific immune responses 19, 20, 67. Based on this mechanism, we employed immunofluorescence staining to detect CRT expressions in CT-26 cells. The strongest CRT signal was observed in the CD@H-RuO_2_ + US group (Figure 5A). As shown in Figure 5B, the highest HMGB1 level was detected in the CD@H-RuO_2_+US group, indicating that the synergistic effects of SDT and CDT mediated by CD@H-RuO_2_ induced the most significant cellular damage. Figure 5C demonstrated that the intracellular ATP level in CT-26 cells treated with CD@H-RuO_2_ under US irradiation decreased compared to the other groups, suggesting that more ATP was secreted into the extracellular region during the combined SDT and CDT treatment achieved through CD@H-RuO_2_. Collectively, these results strongly confirmed that CD@H-RuO_2_ can achieve a robust ICD effect by generating a substantial amount of ROS.

Given that the DAMPs released during the ICD effect induced by CD@H-RuO_2_ can bind to DCs and activate the adaptive immune response 6, 16, we isolated BMDCs from the tibia and femur of BALB/c mice. Immature DCs were obtained by culturing with IL-4 and GM-CSF. Subsequently, the supernatants of CT-26 cells treated under different conditions (control, US, H-RuO_2_, CD@H-RuO_2_, H-RuO_2_+US, CD@H-RuO_2_+US) were collected and co-incubated with immature DCs. The cells were then harvested for flow cytometry analysis to evaluate the expression levels of maturation markers. Notably, co-culturing BMDCs with CT-26 cancer cells pre-treated with "CD@H-RuO_2_ + US" significantly upregulated the expression of maturation markers CD80 and CD86 (Figures 5D, E). This phenomenon indicated that the synergistic effects of SDT and CDT mediated by CD@H-RuO_2_ enhanced DC maturation and thereby induce a robust immune response.

**Figure 6:**
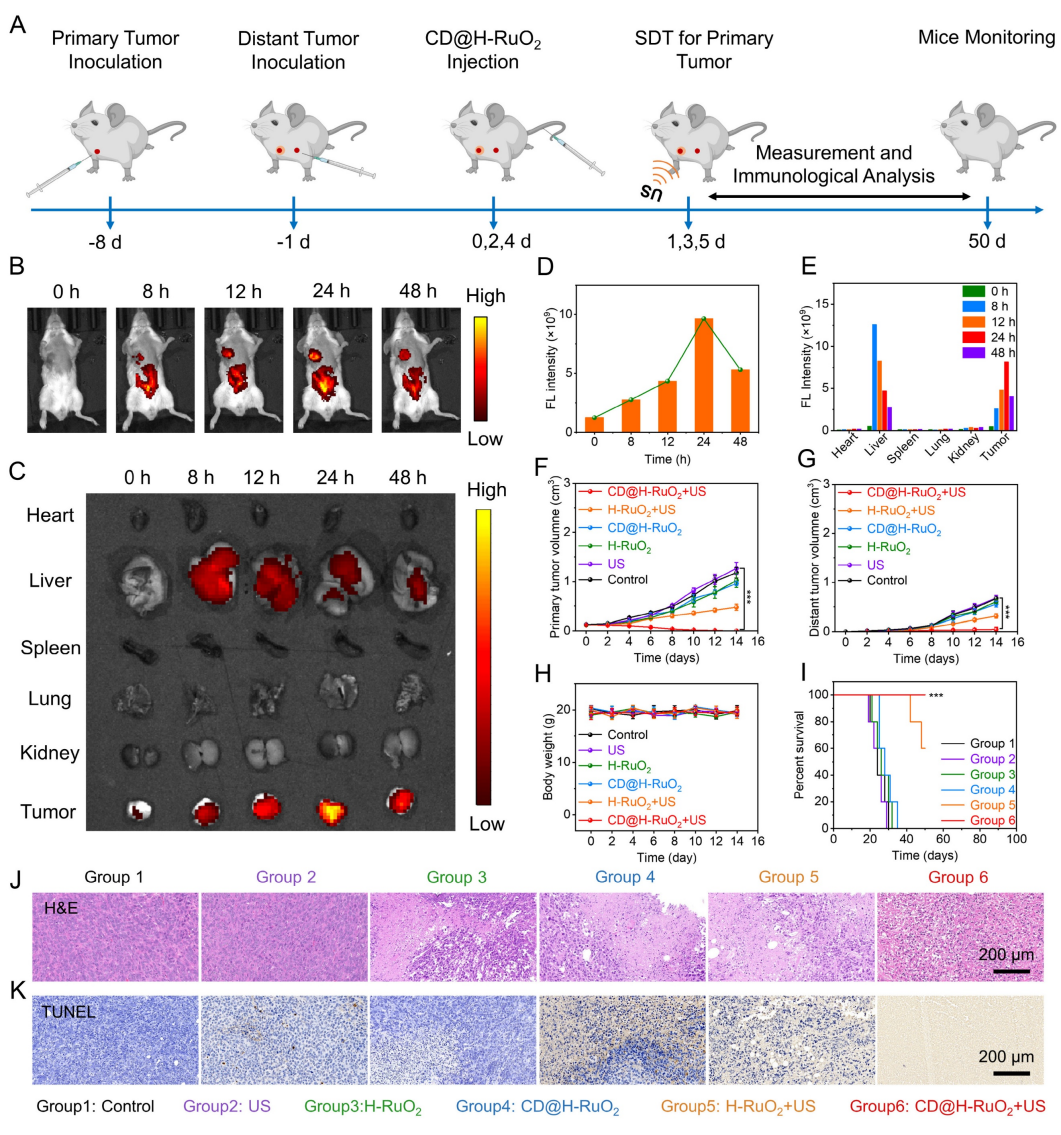
(A) Schematics show the *in vitro* anticancer therapy procedures of CD@H-RuO_2_-mediated combination therapy. (B-E) *In vivo*, *ex vivo* imaging and their quantification at different time points after intravenous injection. (F, G) Primary and distant tumor volume of mice in different groups. (H) Body weight of mice after different treatments. (I) Survival of mice in different groups. (J, K) H&E, and TUNEL staining in different groups. Data are presented as the mean ± SD. (n = 5). ***p < 0.001.

To investigate the *in vivo* antitumor efficacy of CD@H-RuO_2_, we established a bilateral tumor model in mice and administered the treatment regimen as shown in Figure 6A. First, ICG was used to label CD@H-RuO_2_ to determine the optimal time window for SDT. Through the animal imaging system, it was observed that the strongest fluorescence signal at the tumor site occurred 24 hours after intravenous injection, indicating this as the optimal timing for US irradiation. This observation confirmed that CD@H-RuO_2_ effectively accumulated at the tumor site via the EPR effect (Figures 6B-E). Subsequently, the antitumor efficacy of CD@H-RuO_2_ was evaluated by measuring the volumes of primary and distant tumors, as shown in Figures 6F-G and S18. or both primary and distant tumors, H-RuO_2_ alone and CD@H-RuO_2_ alone exhibited weak inhibitory effects on tumor growth. However, with the introduction of US, tumor growth was significantly suppressed, particularly in the CD@H-RuO_2_ + US group. Notably, the proximal tumor achieved complete eradication after three intravenous injections combined with three ultrasound treatments, while the growth of the distal tumor was also markedly inhibited. These results indicated that the increased production of ROS further amplifies the immune response, thereby achieving the best therapeutic outcome. During the *in vivo* treatment, there was no significant difference in body weight between the treated groups and normal mice (Figure 6H), demonstrating the good biocompatibility of CD@H-RuO_2_. Additionally, mice receiving CD@H-RuO_2_ + US treatment survived for over 50 days (Figure 6I), suggesting that the enhanced SDT mediated by the Z-type heterojunctions of CD@H-RuO_2_, combined with CDT, induced a robust immunotherapy effect and prolonged the lifespan of mice. Subsequently, histological analysis of the tumor tissues was conducted. ROS staining revealed that the ROS signal in the CD@H-RuO_2_ + US group was the highest among all groups (Figure S19), further confirming that CD@H-RuO_2_ successfully accumulated in the tumor tissue and generated a substantial amount of ROS under US irradiation. Furthermore, H&E staining and TUNEL staining showed that the primary tumors in the CD@H-RuO_2_ + US group exhibited the most severe damage (Figures 6J-K). Consistent results were also obtained in the histological analysis of the distant tumors (Figure S20). These findings collectively demonstrated that CD@H-RuO_2_-based tumor treatment induced a strong immune response through the generation of large amounts of ROS.

**Figure 7:**
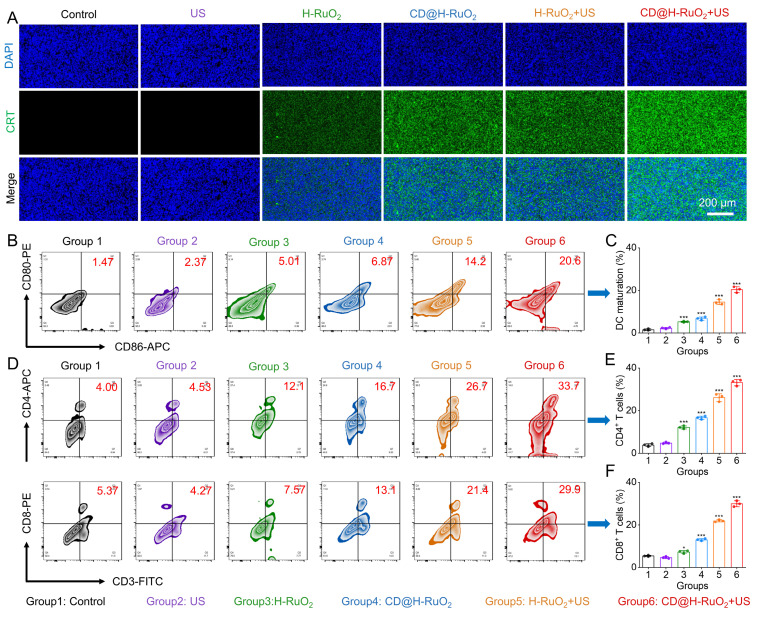
(A) Evaluation of CRT level in tumor tissues after different treatments. (B, C) Measurements of the DC maturation level in the lymph nodes after different treatments. (D-F) T cell activation level in the primary tumor after different treatments including Control, US, H-RuO_2_, CD@H-RuO_2_, H-RuO_2_ + US, and CD@H-RuO_2_ + US. Data are presented as the mean ± SD. (n = 3). *p < 0.05 and ***p < 0.001.

Next, we conducted a comprehensive immunological analysis to elucidate the underlying mechanism of the potent antitumor effect induced by CD@H-RuO_2_. Based on the results of *in vitro* cell experiments, the combination of SDT and CDT mediated by CD@H-RuO_2_ effectively triggered ICD within tumor cells, promoting the release of DAMPs and further enhancing the maturation of DCs. Firstly, the expression of CRT in tumor tissues was evaluated by immunofluorescence staining. As shown in Figure 7A, tumor tissues from the CD@H-RuO_2_ + US group exhibited markedly stronger CRT expression compared to other groups. We further evaluated two additional ICD markers—HMGB1 and ATP—using ELISA and an ATP assay kit, respectively. Consistent with the CRT results, levels of both HMGB1 and ATP were significantly elevated in the CD@H-RuO_2_ + US group relative to other treatments (Figure S21), further confirming the significant induction of ICD by CD@H-RuO_2_ + US. The release of tumor antigens is known to promote DC maturation, which supports the activation of antitumor T cells and adaptive immunity. Therefore, we analyzed immune cell populations in tumor-draining lymph nodes, spleens, and tumor tissues from each group via flow cytometry. Figures 7B, C showed a pronounced increase in the proportion of mature DCs within the tumor-draining lymph nodes of the CD@H-RuO_2_ + US group, confirming the treatment's role in driving DC maturation. As a critical component of the immune response, mature DCs can present tumor antigens to T lymphocytes, promoting their proliferation and activation, and ultimately triggering anti-tumor immune killing effects 18. As expected, after CD@H-RuO_2_ + US treatment, the percentages of CD8^+^CD3^+^ T cells and CD4^+^CD3^+^ T cells in the spleens of mice increased significantly from 5.93% to 23.1% and from 5.96% to 33.5%, respectively, compared to the control group (Figure S22). Subsequently, we evaluated the activation of T cells in tumor tissues. Compared with other groups, the expression levels of CD8^+^CD3^+^ T cells and CD4^+^CD3^+^ T cells in both primary and distant tumors after CD@H-RuO_2_ + US treatment were markedly enhanced (Figures 7D-F and S23). Furthermore, the "CD@H-RuO_2_+US" treatment also significantly reduced the proportion of regulatory T cells (Tregs) in the primary tumor (Figure S24). These results strongly indicate that the construction of heterojunctions enabled cascade amplification of ROS generation, thereby activating the antitumor immune response.

Finally, we evaluated the biosafety of the CD@H-RuO_2_ + US treatment. The major organs of mice subjected to different treatments were analyzed by H&E staining. The results showed no significant differences compared with the control group, and no obvious damage was observed (Figure S25). blood biochemical analysis (Figure S26A) and hematological parameters (Figure S26B) indicated that all blood indicators of the mice remained within the normal physiological range after treatment. In addition, hemolysis assays confirmed outstanding blood compatibility of the material, with a hemolysis rate below 5% even at a high concentration of 400 μg/mL (Figure S27). We further investigated the *in vivo* biodistribution and excretion pathways of the CD@H-RuO_2_ nanoparticles to clarify its metabolic behavior in the body. As shown in Figure S28A, 24 hours after intravenous injection, the CD@H-RuO_2_ mainly accumulated in the liver, spleen and tumor, which was attributed to the capture by the reticuloendothelial system. Over time, by the 14th day after injection, the Ru signal in these organs and tumor tissues significantly decreased, indicating that CD@H-RuO_2_ was almost completely cleared. To determine the excretion pathways, the Ru signal levels in urine and feces were also quantified. Over time, the Ru signals in feces and urine gradually decreased to almost undetectable levels, indicating that CD@H-RuO_2_ was mainly cleared from the mice through the hepatic and renal excretion pathways (Figure S28B). These findings collectively confirm the excellent biocompatibility and biosafety of CD@H-RuO_2_.

## Conclusions

In summary, we report for the synthesis of H-RuO_2_ using Cu_2_O nanospheres as sacrificial templates to achieve efficient SDT and CDT. It is expected that the catalytic activity of H-RuO_2_ nanospheres will be superior to that of solid nanoparticles or nanosheets, which can be attributed to their larger specific surface area and more catalytic active sites. By using CDs as the auxiliary sonosensitizers, we prepared CD@H-RuO_2_ heterojunction nanozymes, which exhibited augmented sonodynamic and multienzyme-mimic activities due to the improved electron hole separation kinetics. The heterojunction construction, along with GSH depletion and hypoxia relief, led to a cascade amplification of antitumor immune response via CD@H-RuO_2_ nanozymes, ultimately triggering a robust ICD. The combination of CD@H-RuO_2_-mediated SDT and CDT co-amplified immunotherapy has displayed significant antitumor effects, resulting in the eradication of primary tumors and the inhibition of distant tumor growth.

## Supplementary Material

Supplementary methods, figures and table.
