# Supplementary material for: Hollow RuO2 nanozymes sensitized by carbon dot sonosensitizers for sonodynamic/chemodynamic-activated immunotherapy
Source: Theranostics. 2026 Jan 1;16(5):2357–71. doi: 10.7150/thno.125880 (PMC12712927; doi:10.7150/thno.125880)
Supplement: Supplementary file 1 — Supplementary methods, figures and table. [file thnov16p2357s1.pdf]

Hollow RuO_2 nanozymes sensitized by carbon dot sonosensitizers for sonodynamic/chemodynamic-activated immunotherapy

Ming Cao [a,1], Yanwei Liu [a,1], Zhenlin Zhang [b,1], Jinming Cai [b], Dengyu Pan [b], Bijiang Geng [b,*], Yunsheng Cheng [a,*]

[a] Department of General Surgery, Second Affiliated Hospital of Anhui Medical University, Hefei, Anhui 230601, China

[b] School of Environmental and Chemical Engineering, Shanghai University, Shanghai 200444, China

[1] Equal Contribution.

* Corresponding author.

E-mail addresses: bjgeng1992@shu.edu.cn (B. Geng), chengyunsheng@ahmu.edu.cn (Y. Cheng)

Keywords: carbon dots, hollow RuO_2 nanospheres, heterojunctions, sonodynamic therapy, immunotherapy

Methods

Preparation of $Cu_2O@RuO_2$

We first utilized PVP and Cu $(NO)_3•3H_2O$ to synthesize Cu_2O. The hydrazine hydrate solution was added dropwise to the mixture solution of PVP and Cu $(NO)_3•3H_2O$. After stirring for 1 h, the products were collected by centrifugation and washing. Then, the synthesized Cu_2O was mixed with $RuCl_3•xH_2O$ under stirring. After centrifugation, the obtained precipitate was re-dispersed in DI water. The pH value of the solution was adjusted to 10. Next, the solution was heated at 80 °C for 30 minutes to improve the quality of the H-RuO_2 shell. Finally, the products were collected by centrifugation and washing.

Preparation of CD@H-RuO_2

For the preparation of H-RuO_2, NH_4OH was slowly added into the solution of $Cu_2O@RuO_2$ under continuous stirring. After 1 h, the products were collected by centrifugation and washing. For the preparation of CDs, ICG and BPEI were selected as the precursors. After a microwave reaction, CDs were obtained for the subsequent loading on the surface of H-RuO_2. We then mixed H-RuO_2 and CDs at a mass ratio of $m_{CD}:m_{H\text{-}RuO2}$ = 2:1. After stirring for 24 h, the products were collected by centrifugation and washing.

Sonodynamic, chemodynamic, and GSH depletion performances

For the detection of US-activated ROS generation, DPBF was mixed with CD@H-RuO_2 under US irradiation (50 kHz, 1.0 W/cm^2). During the irradiation period, the absorption of DPBF was measured to calculate the ROS generation efficiency. In addition, we also utilized TEMP as the $^1O_2$ probe to detect the generation of $^1O_2$ using ESR spectrum. For the detection of •OH through Fenton-like reaction, CD@H-RuO_2 was added to NaAc-HAc buffer solutions containing H_2O_2 at different pH. We

then added TMB to the mixture solution and the absorbance of TMB at 652 nm was measured to calculate the •OH generation efficiency. We also utilized DMPO as the •OH probe to assess the generation of •OH using ESR spectrum. For the evaluation of GSH depletion ability, GSH was mixed with CD@H-RuO_2 and DTNB. The absorbance of DTNB at 412 nm was measured to evaluate the GSH consumption performance of CD@H-RuO_2. The oxygen production capacity of CD@H-RuO_2 was assessed using a portable dissolved oxygen meter (JPBJ-608, RME China).

MTT assay

NIH-3T3 and CT26 were obtained from the Cell Bank of the Chinese Academy of Sciences. NIH-3T3 or CT26 cells were seeded into 96-well plates. After overnight incubation, various concentrations of CD, H-RuO_2, and CD@H-RuO_2 samples were added to the wells. Each well was exposed to US irradiation (50 kHz, 1.0 W/cm^2) for 5 minutes, after which the MTT test was performed.

Live/dead, ROS, and JC-1 staining

CT26 cells were seeded in six-well plates and incubated for 24 hours. Following the experimental protocol (control, US, H-RuO_2, CD@H-RuO_2, H-RuO_2+US, CD@H-RuO_2+US), the corresponding materials were added to each well and co-incubated for 24 hours. For live/dead staining, cells from each well were collected and processed according to the instructions provided in the Calcein-AM/PI cell staining kit. For ROS staining, the cells were fixed with 4% paraformaldehyde, stained with DCFH-DA probe. For JC-1 staining, the cells were stained using the JC-1 Mitochondrial Membrane Potential Detection Kit. Finally, we utilized a confocal microscope to obtain staining images.

ICD marker detection and DC maturation evaluation

After seeding into a six-well plate, CT26 cells were treated according to the experimental protocol (control, US, H-RuO_2, CD@H-RuO_2, H-RuO_2+US, CD@H-RuO_2+US). The release of ATP from cells

was quantified using an ATP detection kit. The HMGB1 detection method was similar to that of ATP, except that the supernatants from each well were collected and measured using the HMGB1 detection kit. The expression of CRT was assessed by immunofluorescence staining in the presence of the primary CRT antibody and the Fluor 647-conjugated secondary antibody.

After isolating from the humerus and tibia of mice, BMDCs were incubated in RPMI-1640 medium with the addition of GM-CSF and IL-4. After incubation of 7 days, the suspension of CT26 cells after different treatments was added into the BMDCs for another incubation of 24 h. CT26 cells were treated with control, US, H-RuO_2, CD@H-RuO_2, H-RuO_2 + US, or CD@H-RuO_2 + US. Finally, we utilized flow cytometry to evaluate the DC maturation level in the presence of FITC-anti-mouse CD11c, APC-anti-mouse CD86, and PE-anti-mouse CD80.

Antitumor therapy and immunological analysis

BALB/c mice aged 8 weeks were purchased from Shanghai SLAC Laboratory Animal Co., Ltd. Animal experiments were performed under the authorization of the Institutional Animal Care and Use Committee of Shanghai University (SYXK 2019-0020). Eight and one days prior to treatment, CT26 cells were inoculated into the right and left axillary region of the mice to establish the primary and distant tumor models, respectively. Subsequently, intravenous administration was performed on days 0, 2, and 4, while the corresponding treatments (1: Control; 2: US; 3: H-RuO_2; 4: CD@H-RuO_2; 5: H-RuO_2+US; 6: CD@H-RuO_2+US, ultrasound for 5 minutes, 50 kHz, 1.0 W/cm^2) were administered on days 1, 3, and 5. On day 14, the mice from each group were euthanized, and major organs and blood samples were collected for H&E staining and biochemical analysis. Additionally, tumors were harvested for HE, TUNEL, and DHE staining. Four days after treatment initiation, the mice were sacrificed, and lymph nodes, spleen, and tumor tissues were collected to evaluate DC maturation, T-

cell activation, and ICD markers.

In vivo metabolism study

Following intravenous administration of CD@H-RuO_2, 5-week-old female Balb/c mice (n = 5) were euthanized on days 1, 7, and 14 post-injections, respectively. Major organs were harvested and digested with aqua regia, and the Ru concentration was subsequently quantified using ICP-MS. To investigate the excretion pathway, another group of Balb/c mice (n = 5) was housed in metabolic cages after CD@H-RuO_2 injection to collect feces and urine. The collected fecal and urinary samples were also digested with aqua regia, and the Ru concentration was determined via ICP-MS.

Statistical analysis

All experiments were performed with at least three independent replicates. Data are presented as mean ± standard deviation (SD). Statistical significance between the experimental group and the control group was assessed using the Fisher LSD test. * indicates a statistically significant difference between the experimental group and the control group (***$p < 0.001$, **$p < 0.01$, *$p < 0.05$).

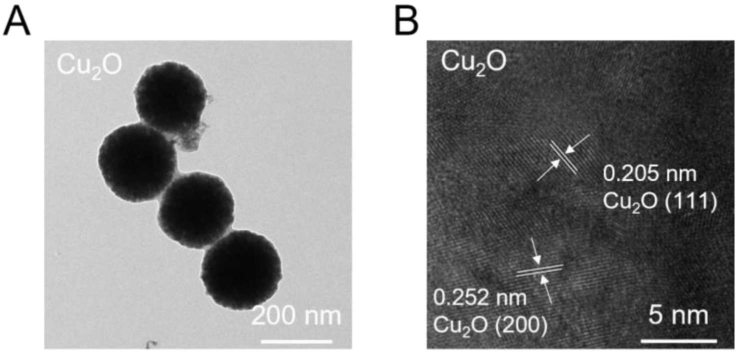

Figure S1. TEM (A) and HR-TEM (B) of Cu_2O.

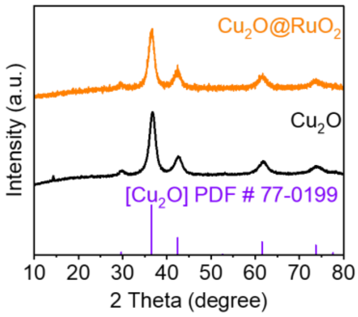

Figure S2. XRD patterns of Cu_2O and $Cu_2O@RuO_2$.

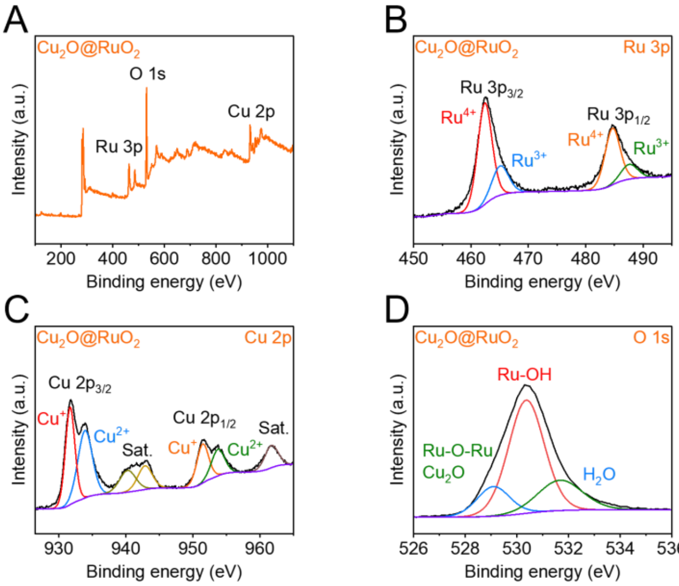

Figure S3. XPS spectra of $Cu_2O@RuO_2$ including survey XPS (A), high-resolution Ru 3p (B), Cu 2p (C) and O 1s (D).

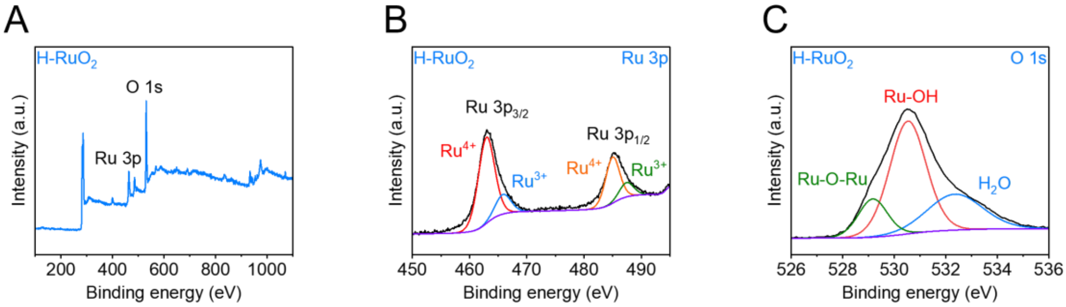

Figure S4. XPS spectra of H-RuO_2 including survey XPS (A), high-resolution Ru 3p (B) and O 1s (C).

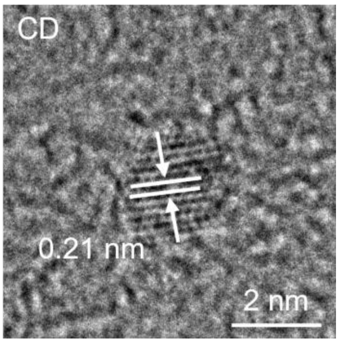

Figure S5. HR-TEM image of CD.

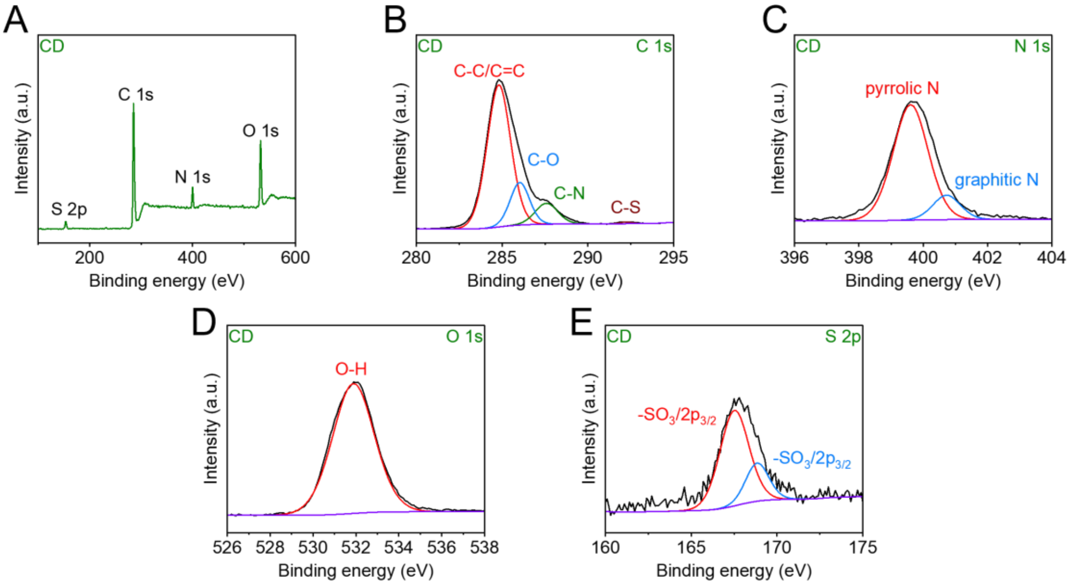

Figure S6. XPS spectra of CD including survey XPS (A), high-resolution C 1s (B), N 1s (C), O 1s (D) and S 2p (E).

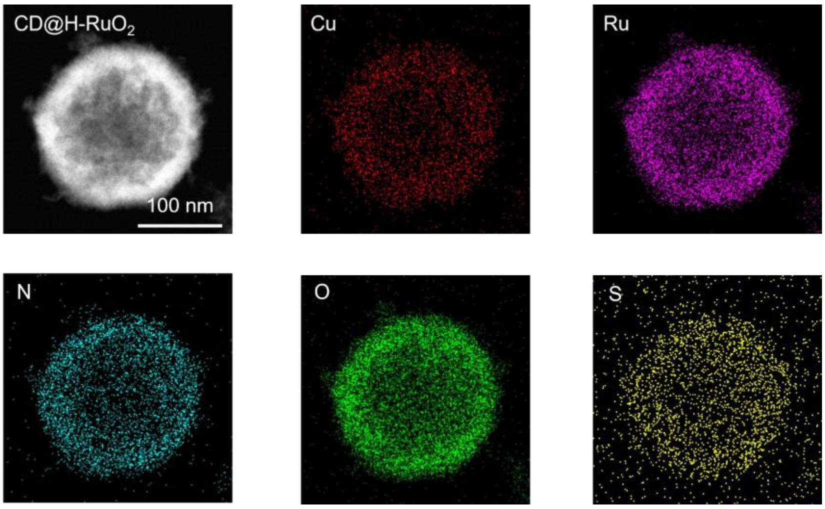

Figure S7. Element mapping analysis of CD@H-RuO_2.

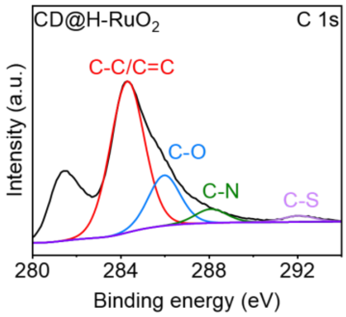

Figure S8. High-resolution C 1s spectrum of CD@H-RuO_2.

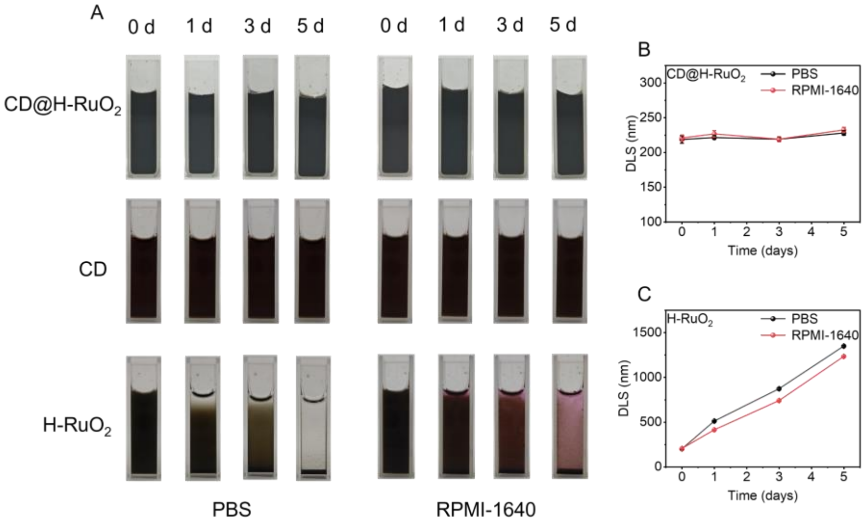

Figure S9. (A) Photographs of CD@H-RuO_2, CD and H-RuO_2 stored for different periods of time at different solution (0, 1, 3, 5 days). (B, C) DLS of CD@H-RuO_2 and H-RuO_2 after storing for different times.

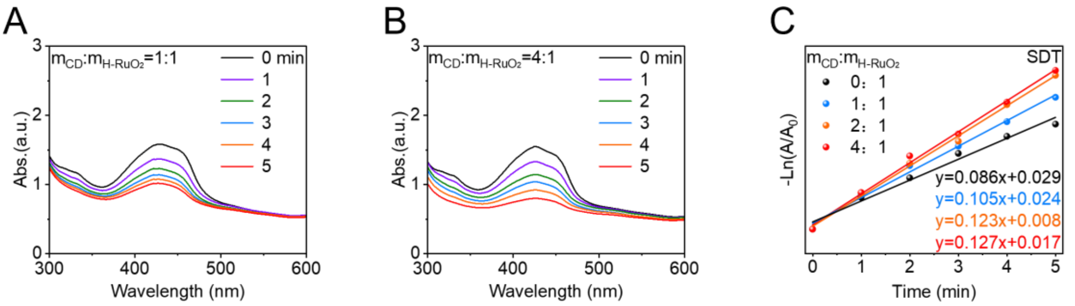

Figure S10. Comparison of the sonodynamic activity of CD@H-RuO_2 prepared at different mass ratios of CD to H-RuO_2.

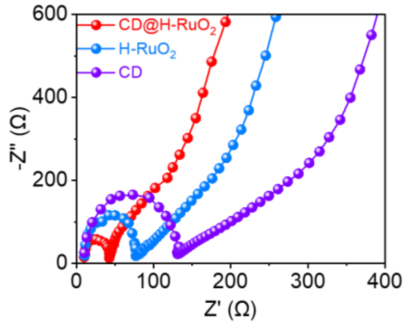

Figure S11. The impedance spectra of CD@H-RuO_2, H-RuO_2 and CD.

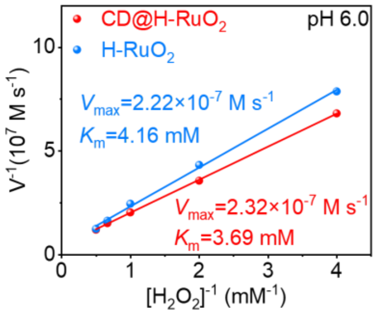

Figure S12. Enzyme catalytic rate constant of CD@H-RuO_2 and H-RuO_2 at pH 6.0.

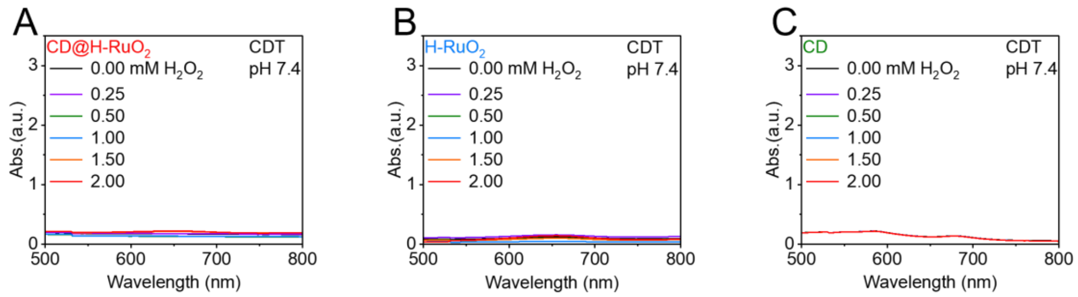

Figure S13. CDT activity of CD@H-RuO_2 (A), H-RuO_2 (B) and CD (C) at pH 7.0.

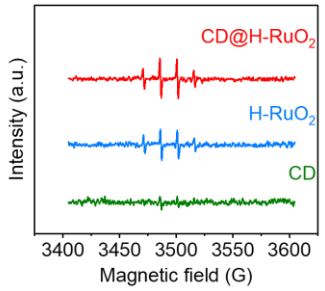

Figure S14. ESR spectra of the •OH generated by CD@H-RuO_2, H-RuO_2 and CD.

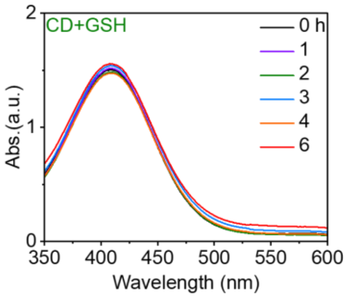

Figure S15. GSH consumption of CD.

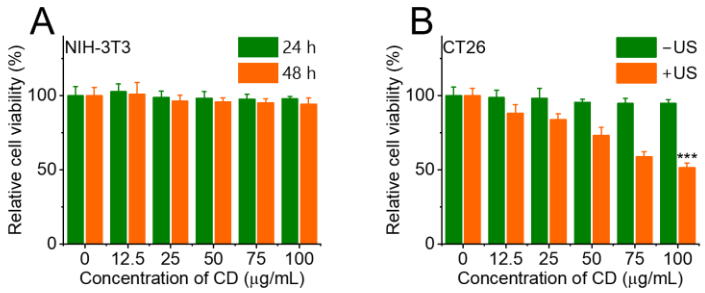

Figure S16. Cytotoxicity evaluation of CD against NIH-3T3 (A) or CT26 cells (B). Data are presented as the mean ± SD. (n = 6). ***$p < 0.001$

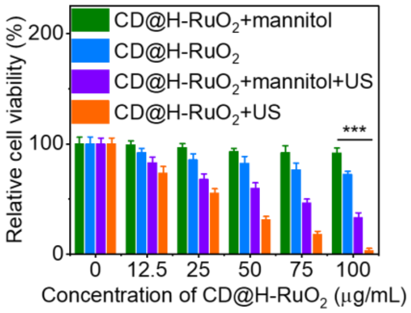

Figure S17. Cell viability of CT26 cells after different treatments. Data are presented as the mean ± SD. (n = 6). ***$p < 0.001$

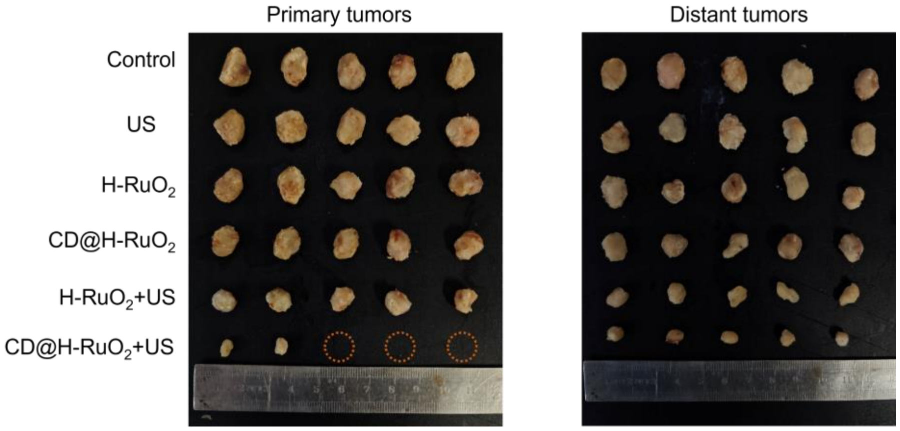

Figure S18. Photographs of the primary and distant tumors in mice after different treatments.

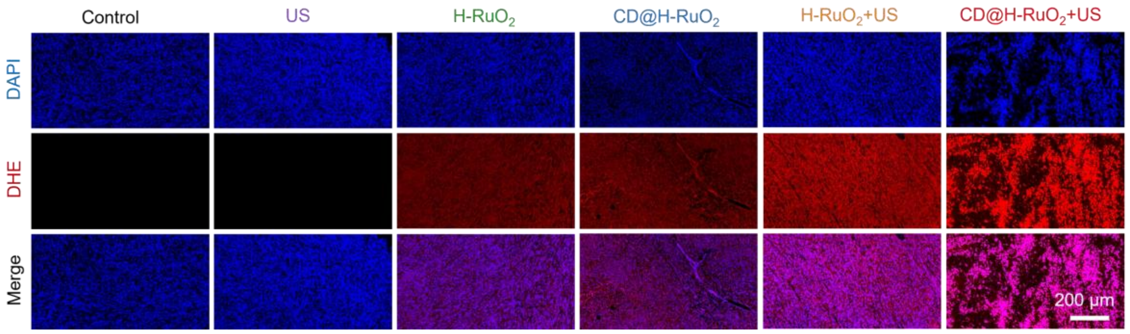

Figure S19. ROS staining of tumor tissue sections following various treatments.

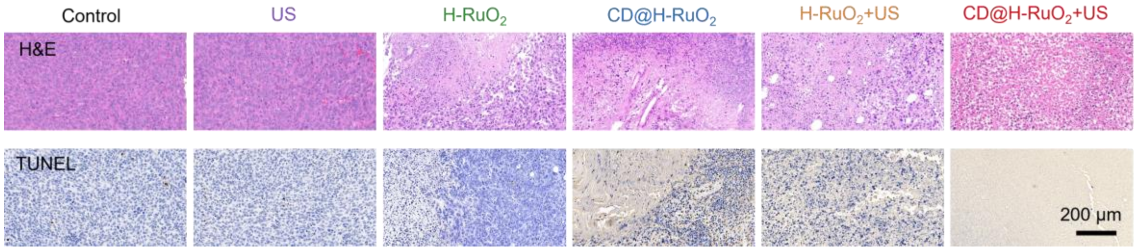

Figure S20. H&E and TUNEL staining of distant tumors after different treatments.

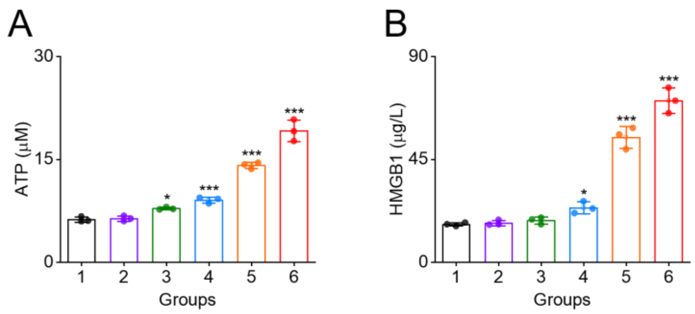

Figure S21. ATP and HMGB 1 level in CT26 tumor tissue after different treatments. Data are presented as the mean ± SD. ($n = 3$). *$p < 0.05$ and ***$p < 0.001$.

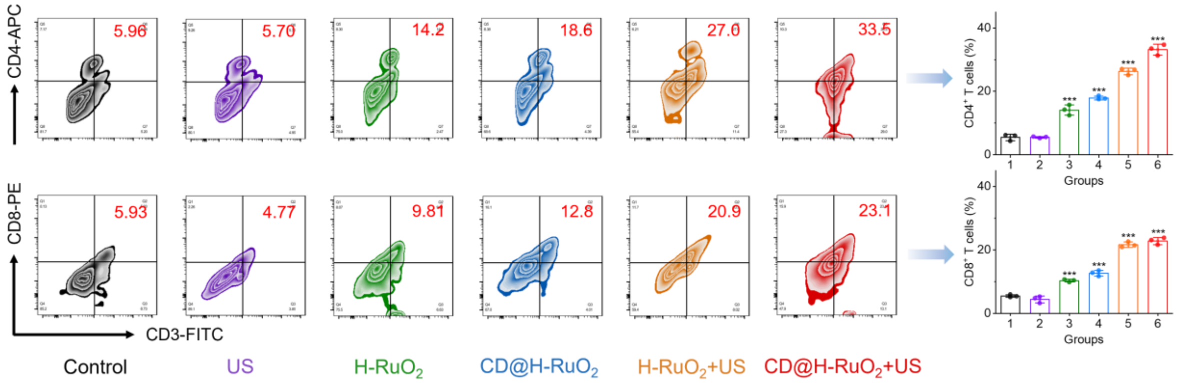

Figure S22. Flow cytometry analysis and the corresponding quantification results of the expression of $CD4^+$ T cells and $CD8^+$ T cells in spleen after different treatments (gated on $CD3^+$ cells). Data are presented as the mean ± SD. (n = 3). ***p < 0.001.

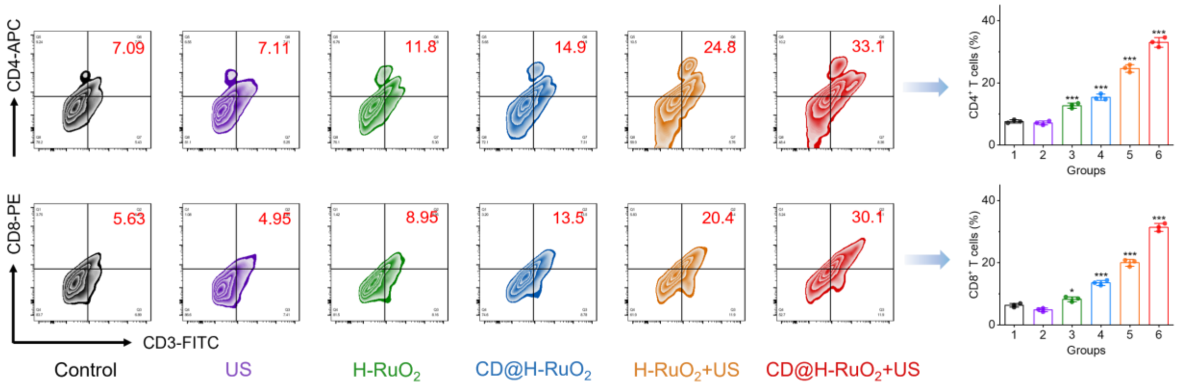

Figure S23. Flow cytometry analysis and the corresponding quantification results of the expression of $CD4^+$ T cells and $CD8^+$ T cells in distant tumor tissues after different treatments (gated on $CD3^+$ cells). Data are presented as the mean ± SD. (n = 3). *p < 0.05 and ***p < 0.001.

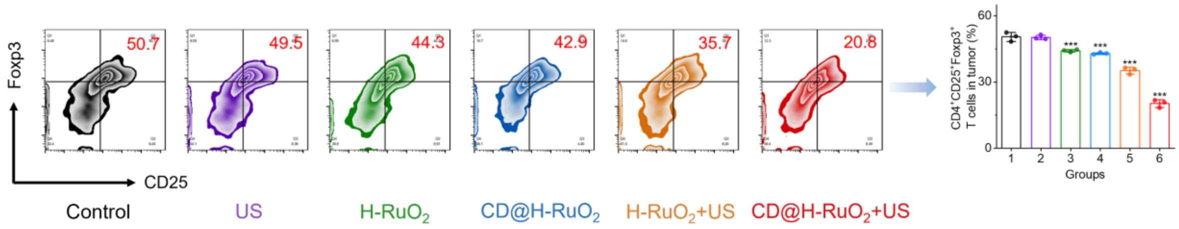

Figure S24. Representative flow cytometric analysis and quantitative data of tumor infiltrating $CD4^+CD25^+Foxp3^+$ Tregs in primary tumors after different treatments. Data are presented as the mean ± SD. (n = 3). ***p < 0.001.

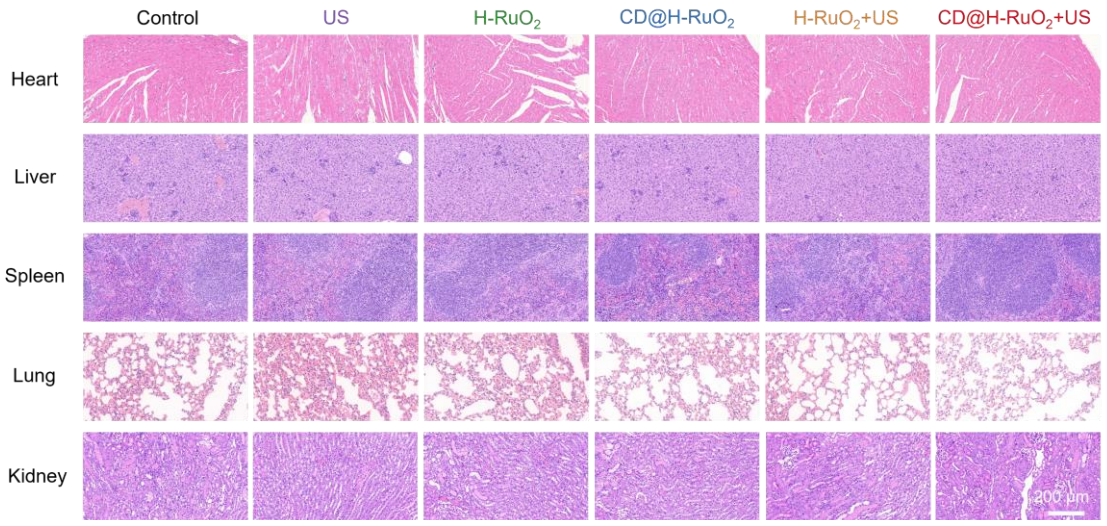

Figure S25. H&E staining of major organs in mice after different treatments.

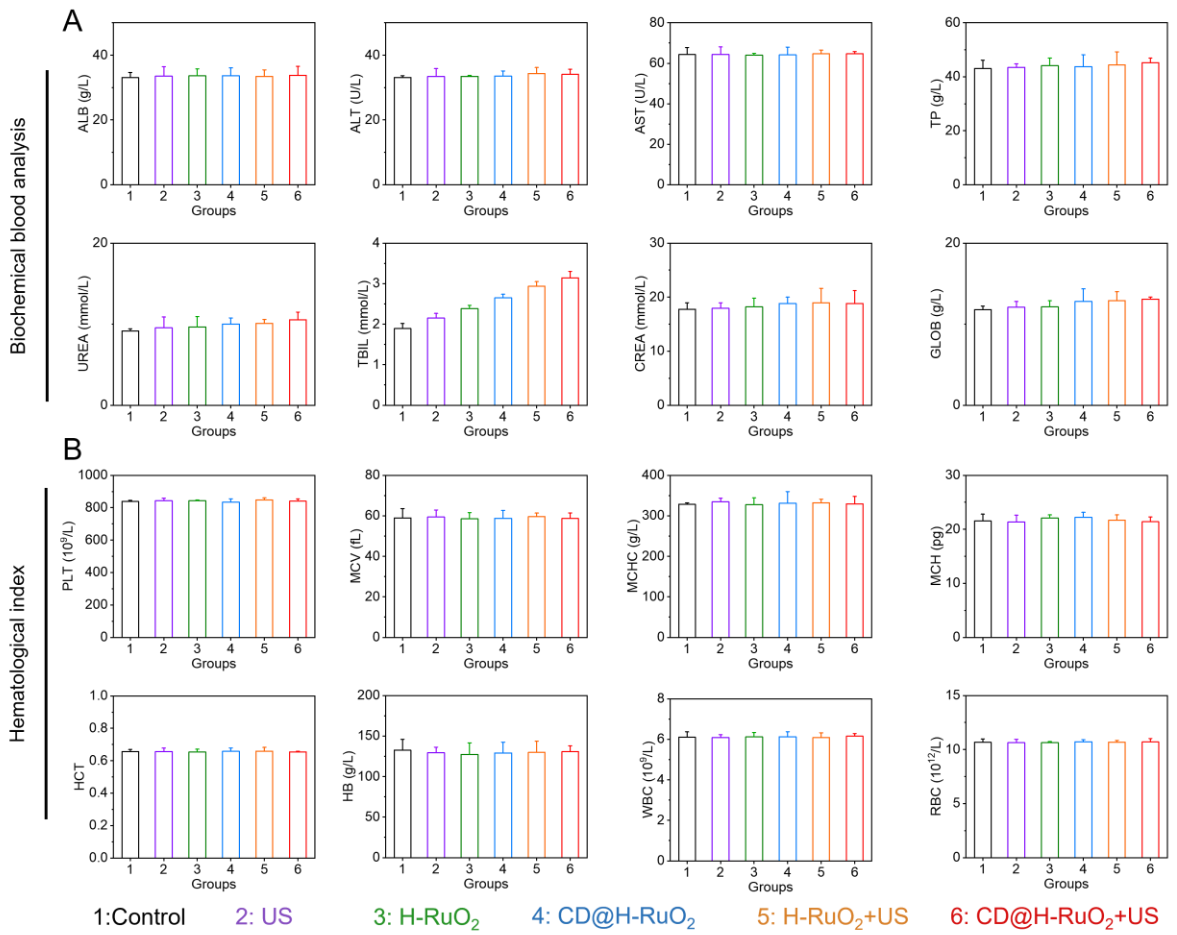

Figure S26. Biochemical blood analysis (A) and hematological index (B) of the mice that were sacrificed at 14 days after different treatments. Data are presented as the mean ± SD. (n = 3).

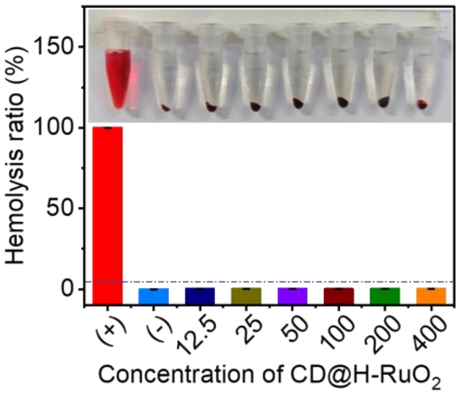

Figure S27. The hemolysis test of CD@H-RuO_2 at different concentration. Data are presented as the mean ± SD. (n = 3).

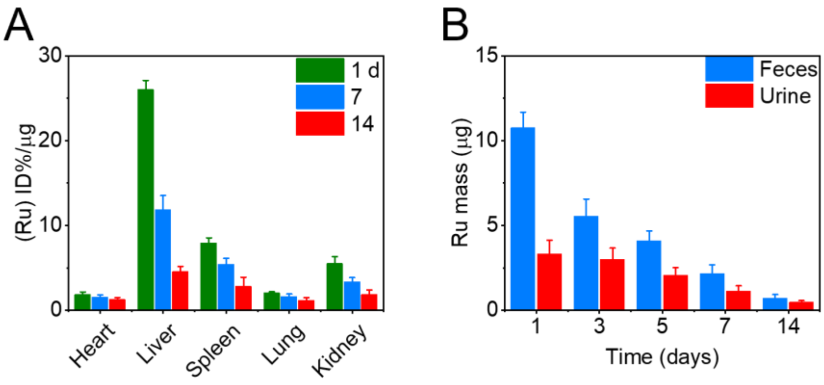

Figure S28. (A) Biodistribution of CD@H-RuO_2 post i.v. injection in mice on different days (n=5 biologically independent samples). (B) The detected CD@H-RuO_2 mass in urine and feces at different time points post i.v. injection of CD@H-RuO_2 (n=5 biologically independent samples). Data are presented as mean values ± SD.

Table S1. Kinetic parameters of different nanozymes with POD-like activity.

Nanozymes	Substrate	V_{max} (10^{-7} M s^{-1})	K_m (mM)
CD@H-RuO_2 (This work)	TMB	2.32	3.69
$PdCu_{0.9}$ [1]	TMB	0.561	57.33
Cu/$CuFe_2O_4$ [2]	TMB	0.865	20.66
$Ti_3C_2T_X$-Pt-PEG [3]	TMB	0.722	34.91
Pd/Cu SAzyme [4]	TMB	0.224	60.62
$CuCo_2S_4$-Pt-PEG [5]	TMB	0.577	33.95
CDs/LDHzyme [6]	TMB	0.617	237.2

References

[1] L.Y. Fang, Y.Y. Zhang, H. Ding, S.K. Liu, J.Y. Wei, L.L. Feng, F. He, S.L. Gai, Y.Z. Dong, P.P. Yang, $PdCu_x$ Bimetallic Nanoalloys with “Hand-in-Hand” Collaboration in POD-like Activity and “Back-to-Back” Confrontation in SPR Effect for Tumor Redox System Control, Adv. Funct. Mater. 34 (2024) 2309338.

[2] W.L. Zhang, D. Sun, B. Yang, J.Q. Liu, Y.L. Yuan, D.Z. Li, Y.L. Sun, G.Q. Guan, Y.L. Liu, D.P. Wu, H. Liao, J.T. Lin, J.Q. Hu, Progressive-composition-transformation-induced enhanced hyperthermia-based nanozyme towards long-lasting and complete therapy of cancer, Chem. Eng. J. 507 (2025) 160645.

[3] Y.L. Zhu, Z. Wang, R.X. Zhao, Y.H. Zhou, L.L. Feng, S.L. Gai, P.P. Yang, Pt Decorated $Ti_3C_2T_x$ MXene with NIR-II Light Amplified Nanozyme Catalytic Activity for Efficient Phototheranostics, ACS Nano 16 (2022) 3105−3118.

[4] X.W. Guan, X.Y. Ge, H.L. Dong, J.J. Wei, J.O. Yang, N. Na, Ultrathin 2D Pd/Cu Single-Atom

MOF Nanozyme to Synergistically Overcome Chemoresistance for Multienzyme Catalytic Cancer Therapy, Adv. Healthcare Mater. 12 (2023) 2301853.

[5] M.S. Xu, R.X. Zhao, B. Liu, F. Geng, X.D. Wu, F. Zhang, R.F. Shen, H.M. Lin, L.L. Feng, P.P Yang, Ultrasmall copper-based nanoplatforms for NIR-II light-triggered photothermal/ photodynamic and amplified nanozyme catalytic therapy of hypoxic tumor, Chem. Eng. J. 491 (2024) 151776.

[6] Z.D. Wang, J.C. Liu, M. Feng, K. Song, K.T. Li, W.D. Liu, S.Y. Guan, Y.J. Lin, ROS-activated selective fluorescence imaging of cancer cells via the typical LDH-based nanozyme, Chem. Eng. J. 470 (2023) 144020.
